# Is Robotic Surgery the Future for Resectable Esophageal Cancer?: A Systematic Literature Review of Oncological and Clinical Outcomes

**DOI:** 10.1245/s10434-024-15148-5

**Published:** 2024-03-13

**Authors:** Nikhil Manish Patel, Pranav Harshad Patel, Kai Tai Derek Yeung, David Monk, Borzoueh Mohammadi, Muntzer Mughal, Ricky Harminder Bhogal, William Allum, Nima Abbassi-Ghadi, Sacheen Kumar

**Affiliations:** 1 Department of Upper GI Surgery, The Royal Marsden NHS Foundation Trust, London, UK; 2 The Upper Gastrointestinal Surgical Oncology Research Group, The Institute of Cancer Research, London, UK; 3 Department of Surgery and Cancer, Imperial College London, London, UK; 4 Department of Upper Gastrointestinal Surgery, Digestive Disease and Surgery Institute, Cleveland Clinic London Hospital, London, UK; 5 Department of Upper GI Surgery, Royal Surrey NHS Foundation Trust, Guildford, Surrey UK

**Keywords:** Esophageal cancer, Robotic surgery, Minimally invasive surgery, Esophagectomy, Lymphadenectomy, Perioperative therapy, Clinical outcomes, Oncological outcomes

## Abstract

**Background:**

Radical esophagectomy for resectable esophageal cancer is a major surgical intervention, associated with considerable postoperative morbidity. The introduction of robotic surgical platforms in esophagectomy may enhance advantages of minimally invasive surgery enabled by laparoscopy and thoracoscopy, including reduced postoperative pain and pulmonary complications. This systematic review aims to assess the clinical and oncological benefits of robot-assisted esophagectomy.

**Methods:**

A systematic literature search of the MEDLINE (PubMed), Embase and Cochrane databases was performed for studies published up to 1 August 2023. This review was conducted according to the Preferred Reporting Items for Systematic Reviews and Meta-Analyses (PRISMA) protocols and was registered in the PROSPERO database (CRD42022370983). Clinical and oncological outcomes data were extracted following full-text review of eligible studies.

**Results:**

A total of 113 studies (*n *= 14,701 patients, *n *= 2455 female) were included. The majority of the studies were retrospective in nature (*n *= 89, 79%), and cohort studies were the most common type of study design (*n *= 88, 79%). The median number of patients per study was 54. Sixty-three studies reported using a robotic surgical platform for both the abdominal and thoracic phases of the procedure. The weighted mean incidence of postoperative pneumonia was 11%, anastomotic leak 10%, total length of hospitalisation 15.2 days, and a resection margin clear of the tumour was achieved in 95% of cases.

**Conclusions:**

There are numerous reported advantages of robot-assisted surgery for resectable esophageal cancer. A correlation between procedural volume and improvements in outcomes with robotic esophagectomy has also been identified. Multicentre comparative clinical studies are essential to identify the true objective benefit on outcomes compared with conventional surgical approaches before robotic surgery is accepted as standard of practice.

**Supplementary Information:**

The online version contains supplementary material available at 10.1245/s10434-024-15148-5.

Surgical resection is a key component of curative management of esophageal cancer, the seventh most common cancer worldwide, and is associated with significant morbidity and mortality.^1^ Neoadjuvant chemo(radio)therapy provides a survival advantage over surgery alone, with 5 year survival rates of up to 50%.^2^ The physical trauma of open esophagectomy with associated postoperative morbidity has considerable impact on survival and health-related quality of life (QOL).^3–5^

Minimally invasive surgery (MIS) confers several benefits to patients with resectable esophageal cancer, with multiple trials comparing outcomes with open surgery.^6–9^ Advantages include reduced postoperative pain due to smaller incisions, lower incidence of pneumonia, and earlier mobilisation, without impacting overall survival (OS) and disease-free survival (DFS).^10–12^ However, evidence suggests that open esophagectomy is associated with shorter operative time but equivalent oncological outcomes to MIS.^13^

Robotic surgical platforms seek to improve perioperative outcomes and enhance what can be achieved with conventional MIS.^14^ The three-dimensional view and articulated instruments afforded by the robotic platform can enhance dissection around difficult planes and improve surgeons’ views.^15^ Robotic surgery is popular in colorectal surgery and gynaecology, and is the gold standard for prostatic resection.^16^

The first reported robot-assisted esophagectomy, using the daVinci telemanipulator instrument (Intuitive Surgical, Mountainview, CA, USA), was published by Melvin et al.^17^ in 2002. Since then, the market for robotic surgical platforms has expanded with numerous systems, including the Hugo^TM^ (Medtronic, Minneapolis, MN, USA) and Versius (CMR Surgical Ltd, Cambridge, UK).

Although the number of robotic esophagectomies has increased worldwide, this procedure is not considered standard treatment for resectable esophageal cancer due to high costs and limited high-level evidence supporting its use.^18^ Current practice may incorporate open surgery and MIS into a ‘hybrid’ procedure. For example, laparoscopy is used for the abdominal phase and an open thoracotomy is used for the chest phase.^19^ This affords patients some of the benefits of MIS, particularly regarding pain and length of hospitalisation.

The primary aim of this systematic literature review is to assess clinical and oncological outcomes of robot-assisted esophagectomy. We describe current trends in practice, evaluate the advantages and disadvantages conferred by the robotic surgical platform, and elucidate evidence of a learning curve among centres who have recently adopted this technique for resectable esophageal cancer.

## Methods

### Search Strategy

This systematic review was conducted according to the Preferred Reporting Items for Systematic Reviews and Meta-analyses (PRISMA) protocols in observational studies and randomised trials,^19^ and was registered on the international prospective register of systematic reviews (PROSPERO), registration number CRD42022370983. A review protocol was not prepared.

A search of the MEDLINE (PubMed), Embase and Cochrane databases was performed by two authors (NMP and PHP), identifying all studies published up to 1 August 2023. The Medical Subject Heading (MeSH) terms ‘robotic surgery’, ‘minimally invasive surgery’, ‘esophageal cancer’, and ‘outcomes’ were included. Conference proceedings and articles not published in English were excluded.

### Data Extraction

Two reviewers (NMP and PHP) screened articles independently by title and abstract before reading the full text of eligible studies. Relevant data including demographics and parameters on perioperative outcomes, including lymph node yield (LNY), anastomotic leak (AL) rate and length of stay (LoS), were collated.

### Statistical Analysis

**Fig. 1:**
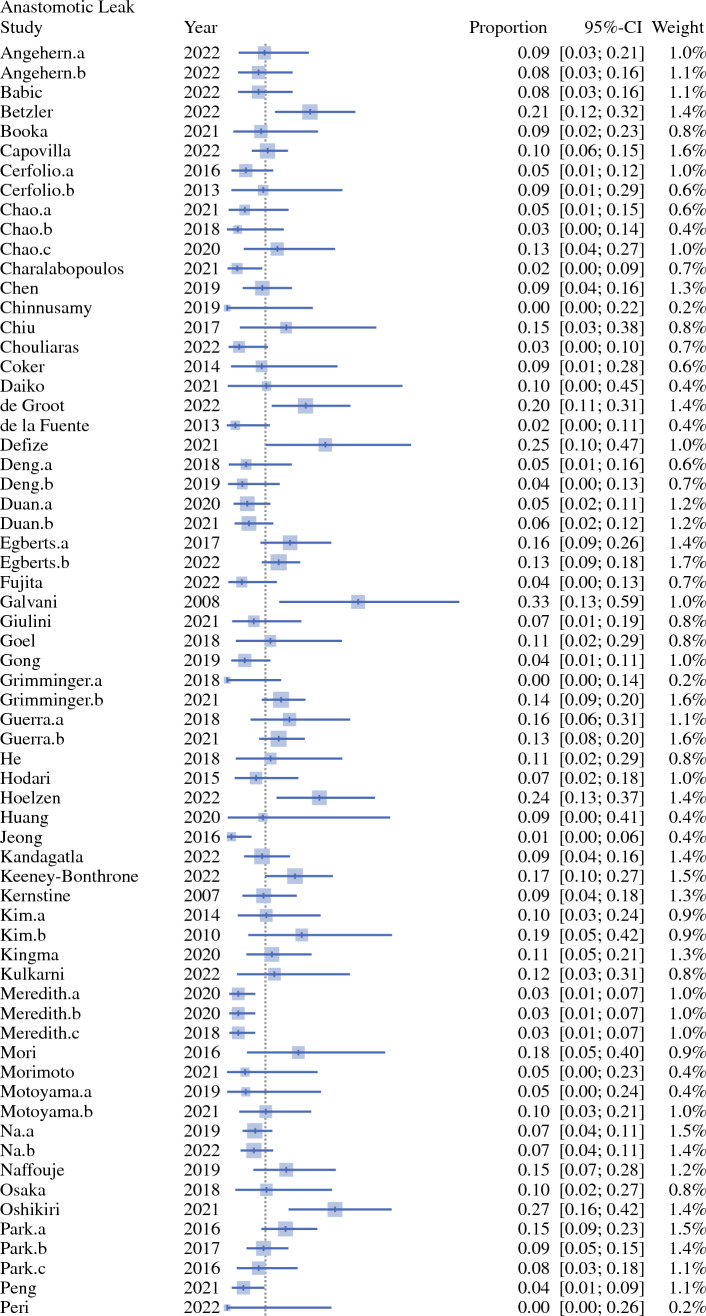

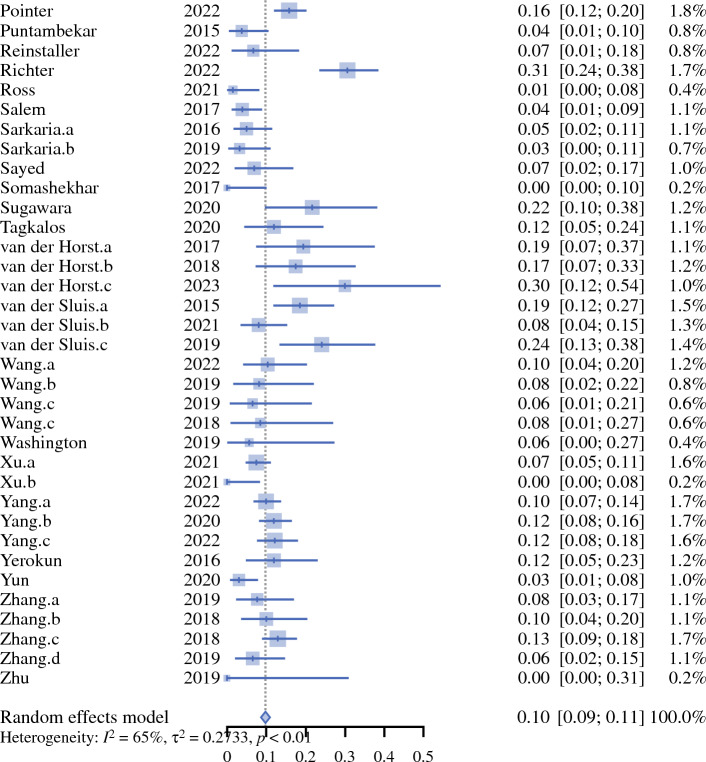
Forest plot on reported anastomotic leak rate. *CI* confidence interval

**Fig. 2:**
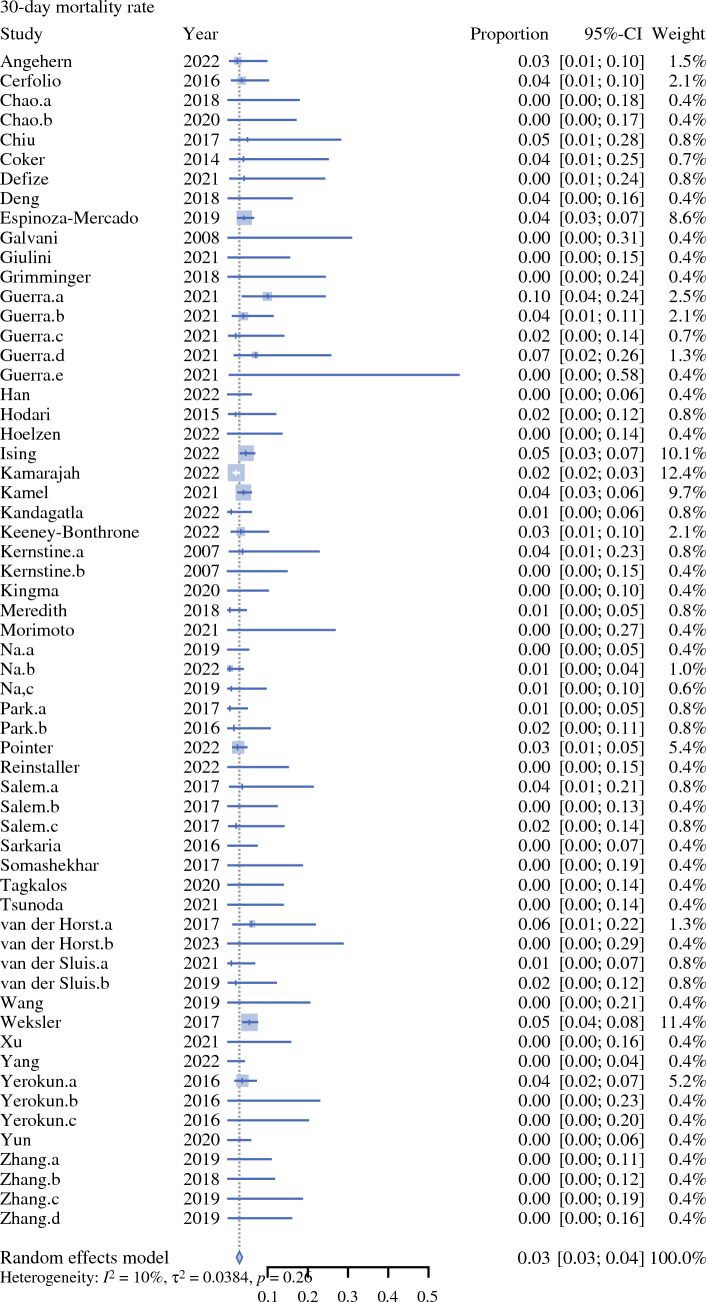
Forest plot on 30 day mortality rate. *CI* confidence interval

**Fig. 3:**
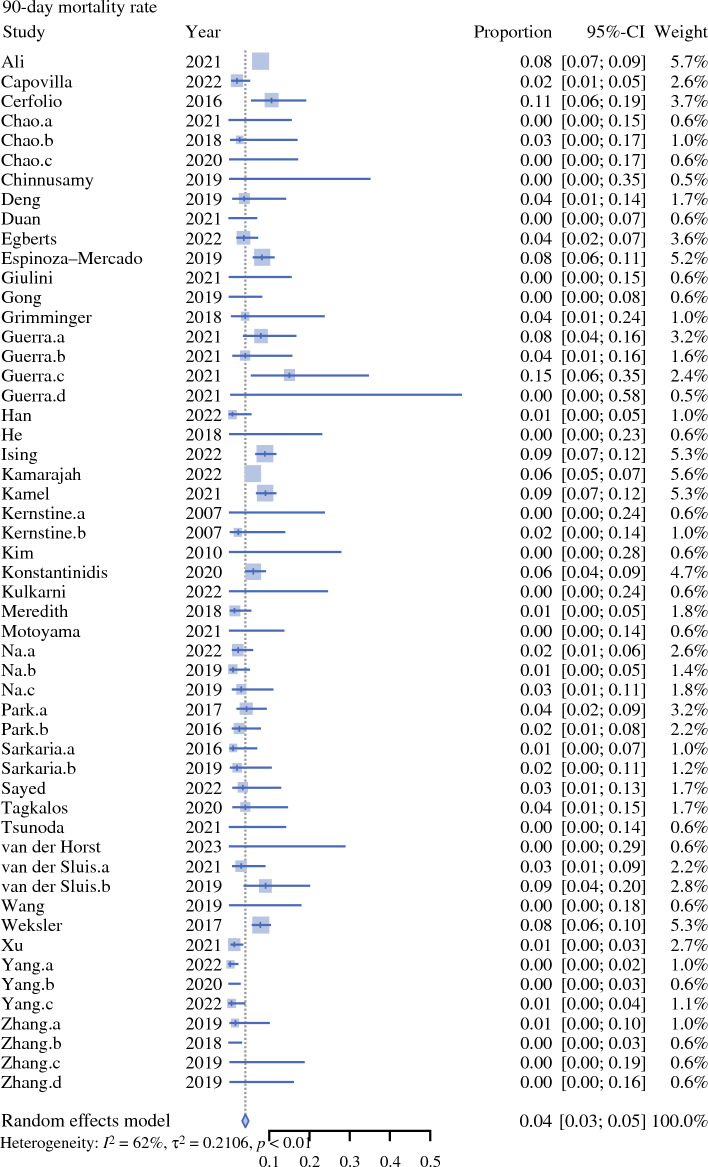
Forest plot on 90 day mortality rate. *CI* confidence interval

Single-arm meta-analyses of oncological and clinical outcomes were performed using RStudio version 4.3.2 (Boston, MA, USA) [Table 1].^20^ Weighted mean (95% confidence interval) and heterogeneity (*I*^2^) were calculated for all studies. Statistical significance was confirmed at *p *< 0.05. Forest plots were constructed for all outcomes, examples of which are demonstrated in Figs. 1, 2 and 3.

**Table 1:** Summary of demographic data from the included studies

		**%**
Total number of included studies	113	
Retrospective/prospective (*n*)	89/24	79/21
Cohort study	90	79
Propensity-matched analysis	18	16
Randomised trial	2	2
Prospective multicentre registry trial	1	1
Population-based analysis	1	1
Case-matched analysis	1	1
Median total number of patients per study (*n*)	54	
Median age of patients (years)	64	
Median number of male/female patients per study (*n*)	44/10	

Total number of each tumour type (*n*)		Median (*n*) per study
Adenocarcinoma	6573	12
Squamous cell carcinoma	5336	22
Other malignancy	300	1

### Bias Assessment

Risk of bias was assessed using the modified Newcastle–Ottawa scale for non-randomised studies, and the modified Jadad scale for randomised trials.^21,22^

## Results

**Fig. 4:**
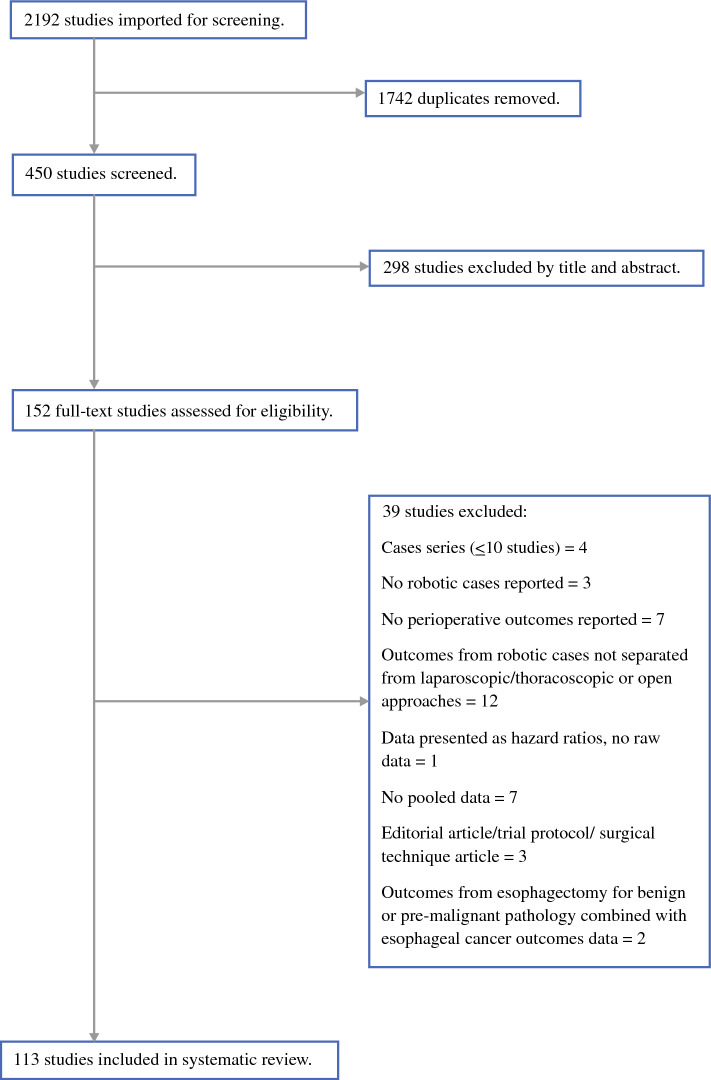
PRISMA reporting standards.^23^ *PRISMA* preferred reporting items for systematic reviews and meta-analyses

The initial literature search yielded 2192 studies. Following screening for full-text eligibility, 113 studies (*n *= 14,701 patients) were included (Fig. 4).

**Fig. 5:**
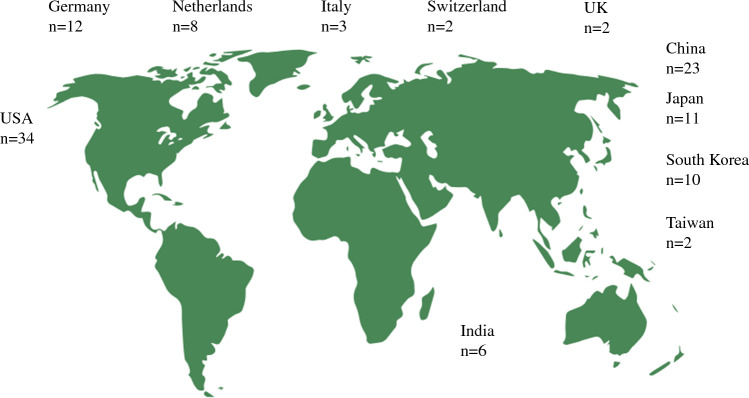
Studies published, by country

Cohort studies reporting on retrospectively collected data were the most common type of study. Four (4%) clinical trials on outcomes following robotic esophagectomy were included. The median number of patients per study was 54, with a median age of 64 years. The most common esophageal malignancy was adenocarcinoma (54%) [Table 1]. Other esophageal malignancies, including gastrointestinal (GI) stromal tumours, were grouped under ‘other malignancy’ (Fig. 5).

Among the included studies, the robotic platform was most commonly used in the thoracic phase (85 studies, 75%) of esophageal cancer resections (Tables 2 and 3). In the abdominal phase, the robotic approach was the most popular (64 studies, 57%), and conventional laparoscopy was used in 20 studies (18%) (Tables 2 and 3). Thirty-one studies (27%) confirmed use of a robotic platform in one phase but did not classify the approach used for others. Most studies reported two-stage procedures (90 studies, 80%) and six reported a transhiatal approach (5%).

Preoperative tumour staging was not presented by the studies. Weighted mean incidence of oncological and postoperative outcomes are presented in Table 4. Use of postoperative opioid analgesia was reported by five studies (4%) [Table 5].

**Table 2:** Surgical approach

Abdomen	Thorax	Neck	No. of studies	%
Robot	Robot	–	63	56
Robot	Thoracoscopic	–	1	1
Robot	Open	–	1	1
Robot	Robot	Robot	2	2
Robot	Robot	Open	7	6
Laparoscopic	Robot	–	22	19
Laparoscopic	Robot	Mediastinoscopic	2	2
Laparoscopic	Robot	Open	8	7
Laparoscopic	Thoracoscopic	–	1	1
Open	Robotic	–	9	8
Open	Robotic	Open	6	5

**Table 3:** Combinations of surgical approaches utilised in the included studies

Abdomen	Chest	*N*^a^
Robotic	Robotic	64
Laparoscopic	Robotic	19
Robotic	Open	3
Open	Robotic	5

^a^The remaining 22 studies reported grouped approaches in their perioperative outcomes, e.g. ‘robotic or laparoscopic abdominal phase’

**Table 4:** Summary of outcomes from the included studies

Outcome	No. of patients	No. of studies	Heterogeneity (*I*^2^) [%]	Weighted mean (95% CI)	*p*-Value
Operative time (minutes)	3619	36	100	372.16 (345.29–399.04)	0
Estimated blood loss (mLs)	3275	31	99	197.71 (167.06–228.36)	0
Critical care length of stay (days)	879	11	89	1.92 (1.28–2.57)	< 0.01
Total inpatient length of stay (days)	3502	22	90	15.18 (14.07–16.29)	< 0.01
Anastomotic leak	8152	70	65	0.10 (0.09–0.11)	< 0.01
Chyle leak	5701	49	65	0.04 (0.03–0.05)	< 0.01
Postoperative pneumonia	7000	80	69	0.11 (0.10–0.13)	<0.01
Lymph node yield	5072	32	100	26.43 (23.36–29.51)	0
R0 rate	12746	82	27	0.95 (0.95–0.96)	< 0.01
30-day mortality	7553	49	10	0.03 (0.03–0.04)	0.26
90-day mortality	9895	45	62	0.04 (0.03–0.05)	< 0.01

*CI* confidence interval

**Table 5:** Perioperative analgesia reported by the included studies

Perioperative analgesia	*N*
Epidural	5
Epidural or intraoperative rectus sheath blocks	1
Patient-controlled analgesia	2
Patient-controlled analgesia + fentanyl skin patch	2
Intraoperative intercostal catheter + intercostal nerve block	1
Not reported	102

Neoadjuvant chemoradiation (70 studies, 62%) was the most frequently used perioperative treatment, followed by neoadjuvant chemotherapy (48 studies, 43%). Use of adjuvant therapy was reported in 14 studies (12.4%).

## Discussion

In this systematic review, we present clinical and oncological outcomes of elective robotic esophagectomy for esophageal cancer. Robotic esophagectomy is a relatively modern modality with variable uptake worldwide. This may contribute to the heterogeneity in results, especially from units with varying surgical experience.

### Type of Study

Retrospective cohort (79%) was the most common study type, with four clinical trials eligible for inclusion.^1,24–26^ Several trials comparing robotic esophagectomy with open and conventional MIS are awaiting publication of the results;^27–29^ therefore, limited data on perioperative outcomes are currently available. Most studies were from North American (30%), and Chinese centres (20%). The wider adoption of robotic surgery throughout the United States reflects greater availability of robotic platforms and supporting infrastructure.^30^ Furthermore, China accounts for nearly half the global disease burden of esophageal squamous cell carcinoma (ESCC), enabling centres to undertake more resections compared with the West.^31,32^

### Surgical Approach

Eighty-five (75%) studies reported a robot-assisted thoracic phase, with 64 studies (57%) performing both robotic abdominal and thoracic phases. A hybrid minimally invasive approach involving laparoscopic abdominal and robotic thoracic phases was reported in 19 studies (17%).^1,27,33^ Open thoracic or abdominal phases were used in combination with robotic surgery in five studies (4%).^34–38^ Initially, the literature reported equivalent oncological outcomes and shorter procedure length in open esophagectomy when compared with thoraco-laparoscopic approaches.^13^ Therefore, many surgeons may lack experience in thoraco-laparoscopic esophageal resection, moving immediately to the robotic console without developing skills in what may be perceived as an ‘intermediate step’ in MIS.^14,34^

Three studies reinforce the notion of learning curves associated with developing proficiency with novel surgical technologies, manifested by analysing learning curves in robotic esophagectomy.^39–41^ These identified the mean number of cases required before surgeons experienced significant improvements in outcomes. Park et al. suggested a change point of 28 robot-assisted esophagectomies for an observed increase in LNY from 25 to 45 (*p *< 0.001);^39^ however, other factors, including marked reduction in the incidence of complications, for example reduction in AL rate, were reported after 80 and 85 cases, respectively. These findings are supported by the cumulative sum (CUSUM) learning curves derived by Kingma et al., where 22 robotic esophagectomy cases were performed before a plateau in estimated blood loss (EBL) and operative time was noticed, suggesting certain components of the procedure take a greater number of cases for expert credentialing.^40^

### Operative Time

Robotic esophagectomy has been associated with longer operating times than open surgery.^1^ This is partly due to time spent ‘docking’ instruments, requiring familiarisation of theatre teams with the robotic platform. This literature review reported a weighted mean operative time of 372.16 min (range 168–808 min) for robotic esophageal cancer resections, taken as the total operating time and not solely time spent on the robotic console.

Kingma et al. identified that after 23 cases, surgeons noticed a reduction in operating time for both the thoracic and abdominal phases, plateauing at case number 70.^40^ Park et al. confirmed that temporal improvement is seen with accumulated experience, but this occurred after 80 cases.^39^ It can be hypothesised that with greater experience comes reduced operating times, reiterating the presence of a learning curve. With sufficient experience, centres may then be able to match higher-volume American and Chinese units.^42–49^

### Perioperative Complications and Length of Stay

An advantage of the robotic platform is the ability to perform finer dissection within challenging anatomical areas, with reduced EBL and rate of visceral injury.^50^ Minimising these complications may allow for shorter recovery times and reduced length of hospitalisation.

### Critical Care and Total Inpatient Length of Stay, Postoperative Pneumonia and Enhanced Recovery After Surgery

There is a significant, multifactorial physiological stress response to major surgery, and open esophagectomy has a significant impact on patients.^5^ The degree of postoperative haemodynamic and respiratory support required typically results in admitting patients to Level 1 care postoperatively.^51^

Weighted mean critical care and total inpatient LoS for the included studies were 1.92 days (range 0.85–23) and 15.2 days (range 7–24), respectively. This indicates significant variation among units, which may be associated with perioperative complications. In their single-centre cohort study of 321 patients, Angehern et al. reported shorter duration of hospitalisation (18.5 days) among their open esophagectomy cohort compared with those who had robotic procedures (20 days, *p *= 0.368).^34^ This contradicts the notion that MIS is associated with shorter LoS. However, given that this is a novel surgical technology, the surgeons may have felt inclined to keep patients in under observation for longer, in anticipation of delayed postoperative complications. This is despite reduced rates of re-intervention among the robotic cohort (5.3%) compared with patients who had open surgery (7.9%).^34^ Pneumonia is a common cause of morbidity after esophagectomy and poor pain control is a major causative factor.^42,52^ Smaller incisions required in robotic esophagectomy and the reduced nerve injury result in less pain after surgery, better respiratory effort and reduced risk of pneumonia. Tsunoda et al. demonstrated a lower rate of pulmonary complications (18%, *p *= 0.006) among patients who underwent robot-assisted esophagectomy compared with conventional minimally invasive esophagectomy (44%).^53^ The incidence of postoperative pneumonia ranged from 0% to 45.4%; however, the literature varied in its definition and criteria influencing treatment decisions.^42^ Notably, Meredith et al. reported no significant difference in the incidence of pneumonia between patients undergoing open, robotic or conventional minimally invasive esophagectomy.^52^ In comparison, three studies reported an incidence of postoperative pneumonia of >30%, despite all patients undergoing laparoscopic and robotic phases, suggesting a multifactorial aetiology for postoperative pneumonia.^33,54,55^

Twenty studies reported total inpatient LoS of < 10 days; none of these studies used an open approach for the thoracic or abdominal phases.^25,41,43,44,52,56–69^ In comparison, 27 studies reported total LoS > 14 days, of which six used an open approach in either the abdominal or thoracic phases.^5,24,25,33,42,45,46,55,70–85^ This indicates the robotic and conventional minimally invasive approaches are associated with shorter LoS, however clinical trials are required to validate this.^1,26,27^

Factors contributing to reduced LoS include less postoperative pain and nausea, earlier introduction of oral intake, and mobilisation.^5,81,86^ Enhanced Recovery After Surgery (ERAS) programmes or ‘fast-track protocols’ in elective upper GI resection have led to improvements in patient outcomes, including LoS and postoperative morbidity, by implementing a standardised pathway for patients and care providers.^87,88^ No studies commented on the use of ERAS. Since robotic esophagectomy is a recent adoption for many units, surgeons may implement a tailored postoperative recovery programme instead of a goal-orientated ERAS pathway.^89^

Twenty-eight studies reported on rate of reoperation, ranging from 0 to 35%. This may reflect varying levels of experience with robotic esophagectomy, and may also be explained by the availability of endoscopy and interventional radiology, which could be used as alternatives to manage selected complications.^28^ Of note, when comparing the open approach with all minimally invasive approaches, the rates of return to theatre did not differ significantly.^34,52,61^

Additional comparative perioperative measures, including time to mobilisation, quantitative data on postoperative pain, and hospital readmission, would be beneficial to describe tangible representative outcomes across studies.

### Blood Loss

Weighted mean EBL across the included studies was 197.7 mLs (range 35–598 mLs), however blood loss per operative phase was not specified. The two studies with the lowest mean blood loss, 35 mLs in total, are also the two where a totally minimally invasive esophagectomy was performed.^63,78^ These studies highlight key advantages offered by MIS through smaller incisions and reduced surgical trauma, giving robotic surgery the advantage over open approaches in resectable esophageal cancer.

### Oncological Outcomes and Perioperative Therapy

Negative resection margins (R0) and LNY were collated to assess perioperative oncological outcomes of the included studies. Aside from reduced postoperative pain and shorter LoS, local disease control must be achieved to potentially improve OS and reduce the chances of recurrence. Although resection margin involvement in the surgical specimen was reported by 87 studies (77.0%), it was not specified whether this related to longitudinal or circumferential margins (CRMs). Median positive margin status from the included studies was 3.48%, demonstrating high rates of ‘curative’ resection were achieved with robotic surgery. In comparison, six studies reported positive resection margin rates of 10% or higher.^40,56,61,90–92^

Comparisons with national registries should be performed for contextualisation. The UK National Oesophago-Gastric Cancer Audit reported a 4.2% positive longitudinal and 20.3% positive CRM status for all esophageal resections performed from April 2018 to May 2021.^93^ Eight years of data from the Dutch Upper Gastrointestinal Cancer Audit (DUCA) reported a positive CRM rate ranging from 3.7 to 6.8%, noting the higher utilisation of neoadjuvant chemoradiotherapy, and 8.7% in Swedish registries.^51,94^ The higher R0 rate reported in this review compared with contemporary registry data, suggests the technical benefits offered by the robotic platform may contribute to greater R0 rates by improving dissection in difficult anatomical locations.^50^ However, other factors, including access to neoadjuvant therapies and disease stage at presentation, may also impact on achieving clear resection margins.

Furthermore, case selection may influence reported outcomes, especially for centres new to performing robot-assisted esophagectomy. Less complex cases may be chosen when testing a novel technique, which may influence outcomes, including R0 resection rate.

None of the included studies reported on CRM status in the resected esophageal specimens. The literature has highlighted the importance of CRM as an independent prognostic factor for local disease recurrence and survival in esophageal cancer.^95–98^ Although the literature suggests that robotic platforms can improve perioperative outcomes, including pulmonary complications and LoS, oncological outcomes are crucial to improving survival for potentially curative disease and should be recorded as standard practice.^36^

Standardised lymphadenectomy is a key component of esophagectomy for accurate disease staging, local disease control and prognostication for OS.^99^ Current guidelines indicate at least 15 LNs must be submitted for pathological examination according to the National Comprehensive Cancer Network (NCCN) and National Oesophago-Gastric Cancer Audit (NOGCA);^93,100,101^ however, standards of and experience in histopathological analysis may vary between centres. This is reflected by the studies included in this review, with a range in LNY of 8–69 nodes, despite a median of 25 nodes. This suggests a significant variability in the extent of lymphadenectomy performed in robotic esophagectomy. Four studies have highlighted that the quality of lymphadenectomy in thoracoscopic esophageal resection is inferior to robotic surgery or thoracotomy, which may explain why uptake of thoracoscopy in the esophagectomy is limited, especially now that robotic surgery is increasingly available.^73–75,82^

Three studies reported significantly lower LNY than the median and the recommended minimum.^14,102,103^ Of these, Washington et al. also reported a positive resection margin rate of 5.56%, above the median of 3.48%.^14^ Furthermore, they reported equivalent LNY when comparing laparoscopic (13.9) and robotic (14.3) esophagectomy.^14^ This was corroborated by Zhang et al., i.e. 19.1 nodes during thoraco-laparoscopic McKeown esophagectomy compared with 19.3 in robotic.^58^ This highlights the importance of following key principles of oncological surgery. In particular, that quality of lymphadenectomy should not be compromised when using a novel surgical technology, even though said new technology may offer other benefits to patients.

Factors influencing the use of oncological therapies include prevalence of different tumour types and recognised standard of care among units. This systematic review highlights international variation in practice, for example, giving definitive chemoradiotherapy in ESCC followed by salvage esophagectomy, versus neoadjuvant chemotherapy followed by surgery.^26,36,46,54,67,70,82,91,92,101^

The published literature supports the use of adjuvant therapy after neoadjuvant treatment and esophagectomy with clear resection margins, citing an improved OS up to 5 years.^104,105^ However, just 14 studies (12%, *n *= 380 patients) reported giving adjuvant therapy. Perioperative therapy, followed by a radical robotic esophagectomy with clear resection margins, without postoperative complications, may enable patients to proceed on to complete adjuvant therapy, improving OS and RFS.^105,106^

### Anastomotic Leak and Chyle Leak

Reported morbidity in esophagectomy can be as high as 50%, with AL and chyle leak (CL) responsible for the greatest risk of prolonged hospitalisation and mortality.^49,107^ Weighted mean reported AL and CL rates were 10% and 4%, respectively.

Fifty-one studies (45%) specified the type of anastomosis created when reporting AL rates. However, AL rate did not vary considerably between circular (8.55%), linear stapled (8.75%) or hand-sewn (8.6%) anastomoses. Six studies reported performing either a robot-assisted hand-sewn or stapled intrathoracic esophagogastric anastomosis, with a leak rate ranging from 0 to 16%.^58,66,90,108–110^ Five studies were carried out in American and Chinese institutions; both were associated with more experience in robotic esophagectomy, which may explain their lower AL rates. In comparison with established national registries, the DUCA reported incidence ranging from 18.2 to 19.3% for all intrathoracic and cervical anastomoses, regardless of technique, and the UK Oesophago-Gastric Anastomosis Audit (OGAA) reported rates of 12.2% and 20.1% for intrathoracic and cervical anastomoses, respectively.^51,111^ As with other outcomes of robotic esophagectomy, volume and experience in performance of the procedural steps directly influence outcomes.

Although most studies reported performing a hand-sewn or stapled extracorporeal anastomosis, there was no appreciable difference in the AL rate between the two subgroups.^38,103,112^ Exteriorising the proximal esophagus and gastric conduit to form a hand-sewn or stapled anastomosis remains the preferred means of restoring continuity as it is technically less challenging than an anastomosis formed entirely within the body cavity through minimally invasive approaches.^83^ Follow-up data from the included studies did not report the incidence of anastomotic strictures and therefore it was not possible to make further comparisons between techniques.

Incidence of CL was reported by 72 studies (64%). Although the average reported rates ranged from 1.1 to 3.8%, the incidence of CL among the included studies was as high as 29%.^33,107^ As suggested by Dezube et al., experience may be the determining factor influencing the risk of CL in esophagectomy. As such, this may be an important parameter to assess for competence in performing robotic esophagectomy in learning curve analyses alongside parameters including operative time.^39,107^ The ramifications of a persistent CL are associated with infection, electrolyte imbalance, hypovolaemia, and nutritional derangement, causing prolonged hospitalisation, delayed oral intake and impact on QOL.

### Comparison of Two- and Three-Stage Robotic Esophagectomy

A total of 51 and 10 studies reported on two- and three-stage esophagectomy using a robotic platform for both abdominal and thoracic phases, respectively. More cases of ESCC were managed with three-stage esophagectomy (25 cases) than two-stage (21 cases), consistent with the preponderance of SCC in the proximal esophagus.^113^ Table 6 highlights that robotic three-stage esophagectomy was associated with longer average operating time, and greater blood loss and AL rate compared with two-stage procedures. The addition of a third phase may explain the prolonged time taken to perform this resection and the associated higher blood loss.

**Table 6:** Comparison of outcomes between two- and three-stage esophagectomy

Median	Two-stage esophagectomy (robotic abdomen and thoracic phases)	Three-stage esophagectomy (robotic abdomen and thoracic phases, open/robotic neck phases)
Studies (*n*)	51	10
Total (*n*) Male/female (*n*)	55 46/11	37 29/8
Adenocarcinoma/SCC/other (*n*)	20/21/1	1/25/0
Age (years)	64	64
Operating time (mins)	387.4	459.55
Blood loss (mLs)	150	200
Anastomotic leak (%)	8.10	12.5
Chyle leak (%)	2.8	2.3
Lymph node yield (*n*)	24	26
R0 (%)	96.4	98.7
Critical care stay (days)	2	2
Total inpatient stay (days)	12.9	11.5
Postoperative pneumonia (%)	9	10.45
30-/90-day mortality (%/%)	0.69/2	0/0

*SCC* squamous cell carcinoma

A robotic cervical phase was reported in four studies, compared with six studies performing an open lymphadenectomy and anastomosis. GI surgeons may begin developing robotic skills by operating within the abdominal cavity, an area more familiar to them given likely previous experience with laparoscopy, before progressing to the thorax. However, uptake of robotic surgery for neck pathology and the cervical esophagus is currently limited according to the published literature.^18,114^ This may explain the greater use of an open approach among the included studies.

In four studies, a stapled esophagogastric anastomosis was formed within the cervical wound to restore continuity of the digestive tract.^42,44,46,115^ In comparison, two studies reported a hand-sewn anastomosis—one formed using the robotic platform and one extracorporeal.^42,115^ The reported AL rate was higher among those who underwent three-stage esophagectomy (12.5%) compared with two-stage (8.10%). This fits with the reported literature, that the incidence of AL is lower in two-stage than three-stage esophagectomy, regardless of whether open, thoraco-laparoscopic or robotic procedures are performed.^116^

### Strengths and Limitations

This is a comprehensive review of outcomes of robotic esophagectomy for resectable esophageal cancer, as evidence by the evaluation of over 100 studies that fulfilled the inclusion criteria. The range of key clinical parameters analysed cover the entirety of the patient’s hospital admission. Assessment of oncological outcomes scrutinises the potential benefits of robot-assisted esophageal resection further by taking into consideration the impact of radical surgery and lymphadenectomy on disease- and recurrence-free survival. Meta-analysis of clinical and oncological outcomes objectively validates the findings of the included studies; however, as demonstrated in Table 2, there was a significant degree of heterogeneity between the included studies in a number of outcomes. Uptake of robotic surgery is not consistent internationally and this is reflected in the reported outcomes. Furthermore, there were only four clinical trials on robotic esophageal cancer resection. These may limit the conclusions that can be drawn from current evidence. Data from prospective trials comparing open, thoraco-laparoscopic and hybrid procedures with robot-assisted esophagectomy are therefore required in order to make more definitive conclusions on the advantages of robotic surgery for resectable esophageal cancer.

## Conclusions

This systematic review presents numerous advantages to perioperative outcomes conferred by robot-assisted surgery for resectable esophageal cancer. We have identified reduced intraoperative blood loss, shorter LoS, and greater LNY as being particularly advantageous. However, it is not yet clear that robotic surgery leads to a difference in survival in resectable esophageal cancer. This review highlights the presence of a learning curve and a minimum number of cases that may need to be performed before noticing marked improvement in postoperative outcomes afforded by robot-assisted surgery. Before standardised adoption of the robotic approach over current techniques, multicentre comparative clinical trials must be undertaken to identify the true objective benefit on perioperative and medium- and long-term outcomes. These may include involvement of longitudinal and circumferential resection margins, return to normal physical activities and work, and QOL, DFS and OS. The latter three should be benchmarked as standardised outcomes to determine whether the robotic platform affords an advantage in patient-reported and oncological outcomes.

### Supplementary Information

Below is the link to the electronic supplementary material.

Supplementary file 1 (DOCX 2182 kb)

## Data Availability

The data that support the findings of this study are available on request from the corresponding author.
