# Supplementary material for: Is Robotic Surgery the Future for Resectable Esophageal Cancer?: A Systematic Literature Review of Oncological and Clinical Outcomes
Source: Ann Surg Oncol. 2024 Mar 13;31(7):4281–97. doi: 10.1245/s10434-024-15148-5 (PMC11164768; doi:10.1245/s10434-024-15148-5)
Supplement: Supplementary file 1 — (DOCX 2182 kb) [file 10434_2024_15148_MOESM1_ESM.docx]

| Author | Year | Number of patients (n) | Adenoca | SCC | Other | Average age (years) | Mean operative time (mins) | Average estimated blood loss (mls) | Anastomotic leak (%) | Chyle leak (%) | Average lymph node yield (n) | R0 (%) | Average length of critical care admission (days) | Average total length of inpatient stay (days) | Post-operative pneumonia (%) | 30-day mortality (%) | 90-day mortality (%) |
| --- | --- | --- | --- | --- | --- | --- | --- | --- | --- | --- | --- | --- | --- | --- | --- | --- | --- |
| Peng | 2021 | 121 | 52 | 67 | 2 | 65 |  |  | 5 |  |  | - | - |  |  | - | - |
| (Reverse-puncture anastomosis) |  | 57 | 26 | 30 | 1 | 64.7 | 232.5 | 204.7 | 3.1 | 0 | 22.4 | 100 |  | 9.49 | 1.6 |  |  |
| (Manual purse-string anastomosis) |  | 64 | 26 | 37 | 1 | 65.6 | 262.3 | 297.6 | 5.3 | 0 | 25.3 | 100 |  | 17.8 | 3.5 |  |  |
| Chao | 2021 | 44 | 2 | 42 | 0 | 57.1 | - | 96.1 | 4.5 | 4.5 | 29.6 | - | 1.68 | 18.1 | 9.1 | - | 0 |
| Kingma | 2020 | 70 | 52 | 13 | 5 | 61.4 | 414 | 275 | 11.4 | 2.86 | 28 | 88.6 | - | 12 | - | 0 | - |
| Park | 2017 | 140 | - | 131 | 9 | 65.1 | 468 | - | 9.29 | - | 41 | 96.4 | - | 14 | 8.57 | 0.71 | 4.29 |
| Zhu | 2019 | 10 | 0 | 10 | 0 | - | 381 | 102.5 | 0 | 0 | 22.4 | 100 | - | - | 0 | - | - |
| Sarkaria | 2016 | 100 | 86 | - | - | 62 | 379 | 250 | 5 | 1 | 24 | 90 | - | 9 | 2 | 0 | 1 |
| Richter | 2022 | 160 | 141 | 15 | 4 | 64 | 329.3 | - | 30.6 | - | - | - | - | 20.5 | 21.9 | - | - |
| Grimminger | 2018 | 25 | 18 | 7 | 0 | 61.5 | 410.2 | - | 0.12 | 0.04 | 24.5 | 92 | 9.7 | 21.8 | 8 | 0 | 4 |
| Meredith | 2020 | 144 | - | - | - | 66 | 409 | 155 | 2.8 | - | 20 | 100 | - | 9 | 6.9 | - | - |
| Yun | 2020 | 130 | 0 | 130 | 0 | 63.7 | 275.6 | 110.8 | 3.08 | 6.15 | 39.1 | 97.7 | 1.08 | 16.5 | 3.85 | 0 | - |
| Meredith | 2020 | 144 | - | - | - | 66 | 409 | 156 | 2.8 | 0.7 | 20 | 100 | - | 10 | 6.9 | - | - |
| Park | 2016 | 62 | - | 62 | - | 64.3 | 490.3 | 462.9 | 8.1 | - | 37.3 | - | - | - | - | 1.6 | - |
| Deng | 2018 | 42 | 0 | 42 | 0 | 60.7 | 354 | 97 | 4.8 | 2.4 | 21.9 | - | - | 14.1 | 7.1 | 0 | - |
| Gong | 2019 | 91 | 0 | 86 | 5 | 60.0 | 318 | 215.5 | 4.4 | 1.1 | 22.8 | 100 | - | 16.6 | 9.89 | - | 0 |
| Chen | 2019 | 101 | 0 | 101 | 0 | 61.9 |  | 115.7 | 8.8 | 2.9 | - | - | 3.8 | 16.7 | 17.6 | - | - |
| Angehern | 2022 |  |  |  |  |  |  |  |  |  |  |  |  |  |  |  |  |
| (Continuously sutured anastomosis) |  | 32 | 28 | 3 | 1 | 68.5 | 467 | - | 9.3 | - | - | 93.7 | 6 | 20 | 3.1 | - | - |
| (Linear-stapled anastomosis) |  | 21 | 20 | 1 | 0 | 70 | 453 | - | 9.5 | - | - | 100 | 7 | 21 | 9.5 | - | - |
| Jeong | 2016 | 88 | - | - | - | - | 288 | 200 | 1.1 | - | - | - | 1.8 | 12 | 3.4 | - | - |
| Deng | 2019 | 52 | - | 52 | - | 61 | 353 | 96.3 | 3.8 | - | 21.5 | - | - | 14.3 | 9.6 | - | 3.8 |
| Espinoza-Mercado | 2019 | 433 | 363 | 70 | - | 64 | - | - | - | - | 17 | 94.9 | - | 9 | - | 4.2 | 8.2 |
| Wang | 2022 |  |  |  |  |  |  |  |  |  |  |  |  |  |  |  |  |
| (Single-docking of robotic console) |  | 45 | 6 | 36 | 3 | 59 | 395 | 100 | 11.1 | - | 19 | 97.8 | 1 | 12 | 11.1 | - | - |
| (Double-docking of robotic console) |  | 23 | 1 | 18 | 4 | 59 | 380 | 90 | 8.7 | - | 17 | 100 | 1 | 11 | 4.3 | - | - |
| Guerra | 2018 | 38 | 29 | 9 | - | 67 | 550 | 80 | 16 | 10 | 33 | 100 | 2 | 10 | - | 10 | - |
| Zhang | 2019 | 66 | 0 | 64 | 2 | 62.3 | 302 | 200 | 7.6 | 0 | 19.2 | 100 | - | 9 | 6.1 | 0 | 1.5 |
| Sarkaria | 2019 | 64 | 59 | 4 | 1 | 61 | 384 | 250 | 3 | 0 | 25 | 96.9 | 5 | - | - | - | 2 |
| Salem | 2017 |  |  |  |  |  |  |  |  |  |  |  |  |  |  |  |  |
| (BMI 18.5-24.99 kg/m^2^) |  | 28 | 22 | 4 | 2 | 66 | 402 | 136 | 0 | - | 22 |  | - | 10 | 0 | 3.6 | - |
| (BMI 25-29.99 kg/m^2^) |  | 56 | 52 | 4 | 0 | 67 | 412 | 162 | 1.8 | - | 21 |  | - | 11 | 3.6 | 0 | - |
| (BMI ≥ 30 kg/m^2^) |  | 45 | 40 | 2 | 3 | 66 | 446 | 174 | 8.9 | - | 20 |  | - | 13 | 8.9 | 2.2 | - |
| Capovilla | 2022 | 204 | 154 | 50 | - | 64.3 | - | 334 | 10.3 | - | - | 94.1 | 3 | 16 | - | - | 1.9 |
| Ross | 2021 | 67 | 64 | 1 | 2 | 69 | 327 | 150 | 1.49 | - | 8 | 98.5 | - | - | 1.49 | - | - |
| van der Horst | 2018 | 40 | 19 | 21 | - | 67 | - | - | 17.5 | 25 | 24 | 98 | 1 | 13 | 27.5 | - | - |
| Motoyama | 2019 | 21 | - | 21 | - | 63 | 634 | 492 | 5 | 5 | 52 | - | - | - | 0 | - | - |
| Kim | 2014 | 40 | - | 40 | - | 63.6 | 428.6 | 156.7 | 10 | 5 | 42.6 | 97.5 | 1 | 14 | 2.5 | - | - |
| Angehern | 2022 | 76 | 71 | 4 | 1 | 69.5 | - | - | 7.9 | - | 24.5 | 96.1 | - | 20 | 30.3 | 2.6 | - |
| Egberts | 2017 | 75 | 72 | - | 3 | 66 | 392 | 172 | 16 | - | 29 | 95.8 | - | 16 | - | - | - |
| Giulini | 2021 | 44 | 36 | 8 | - | 64 | 301 | - | 6.8 | 6.8 | 27 | 95.5 | 7 | 17 | 27.3 | 0 | 0 |
| de Groot | 2022 | 70 | 55 | 11 | 4 | 66 | - | 250 | 20 | 7 | 43 | 99 |  | 12 | 26 | - | - |
| Oshikiri | 2021 | 51 | 6 | 45 | - | 67 | 808 | 35 | 27.5 | - | - | - | - | 24 | 17.6 | - | - |
| Duan | 2020 | 118 | - | 118 | - | 59.1 | 310 | 200 | 5.08 | 0.85 | 23 | 100 | 8 | 18.2 | 11 | - | - |
| de la Fuente | 2013 | 50 | 46 | 3 | 1 | 66 | 445 | 146 | 2 | 4 | 20 | 100 | 2 | 9 | 10 | - | - |
| Zhang | 2018 | 61 | 0 | 58 | 3 | 61.6 | 315.6 | 189.3 | 9.8 | 1.6 | 19.3 | 100 | - | 10 | 6.6 | 0 | - |
| Charalabopoulos | 2021 | 80 | 79 | 0 | 1 | 64.6 | 420 | - | 2.5 | 1.25 | 30 | 87.5 | - | 14 | 8.75 | - | - |
| Wang | 2019 | 37 | 9 | 26 | 2 | 62.7 | 340 | 120 | 8.1 | 2.7 | - | - | - | 10 | 8.1 | - | 0 |
| Han | 2022 | 124 | - | 117 | 8 | 62.8 | 293.5 | 173.7 | - | - | 19.6 | - | - | 9 | - | 0 | 0.8 |
| Sugawara | 2020 | 37 | 3 | 34 | 0 |  | - | - |  | - |  | - | - | - | - | - | - |
| (Transthoracic oesophagectomy) |  | 19 | 3 | 16 | 0 | 69 | - | - | 21 | - | 55 | - | - | - | - |  |  |
| (Transmediastinal oesophagectomy) |  | 18 | 0 | 18 | 0 | 66 | - | - | 22 | - | 69 | - | - | - | - |  |  |
| Kandagatla | 2022 | 112 | 98 | 3 | 11 | 64.1 | 357 | 64.6 | 8.5 | 1.9 | 19 | 100 | - | - | 10.4 | 0.9 | - |
| Xu | 2021 | 310 | 0 | 310 | 0 | 64.86 |  |  | 7.4 | 1.3 | 22.46 | 100 | - | - | 8.7 | - | 1.3 |
| Mehdorn | 2020 | 12 | 12 | 0 | 0 | 61.5 | 357.8 | - | - | - | 31 | 91.7 | - | 18.9 | - | - | - |
| Tsunoda | 2021 | 49 | 2 | 46 | 1 | 68 | 712 | 80 | - | - | 60 | 94 | - | - | 18 | 0 | 0 |
| Motoyama | 2021 | 51 | 6 | 43 | 2 | 65 | 646 | 407 | 10 | - | 52 | 100 | - | - | 16 | - | 0 |
| Duan | 2021 | 109 | 0 | 109 | 0 | 60 | 321.2 | 198.1 | 5.5 | 0.9 | 24.8 | 100 | 5.5 | 18 | 0.9 | - | 0 |
| Chao | 2018 | 37 | 0 | 37 | 0 | 58.6 | - | 94.05 | 2.7 | - | 37.02 | 100 | - | 17.5 | 8.1 | 0 | 2.7 |
| Ising | 2022 | 527 | 450 | 77 | 0 | 63.8 | - | - | - | - | 16.7 | 96.4 | - | 12.7 | - | 4.6 | 8.9 |
| Yerokun | 2016 | 231 | 186 | 45 | 0 | 64 | - | - | - | - | 16 | 93.5 | - | 10 | - | 3.7 | - |
| (Grasper retraction) |  | 27 | 0 | 27 | - | 58.7 | - | - | 15 | - | 10 | - | - | - | 15 | 0 | - |
| (Thread retraction) |  | 32 | 0 | 32 | - | 63.3 | - | - | 9 | - | 10 | - | - | - | 9 | 0 | - |
| Wang | 2019 | 31 | 8 | 22 | 1 | 59.7 | 387.4 | 110 | 6.5 | - | 17.1 | 96.8 | 1 | 12 | 3.23 | 0 | - |
| Grimminger  (Totally Robot-assisted minimally invasive esophagectomy) | 2021 | 175 | 142 | 27 | 6 | 61 | 385 | - | 13.6 | 2.5 | 28 | 93.7 | - | 13 | 15.3 | - | - |
| (Hybrid Robot-assisted minimally invasive esophagectomy) | " | 67 | 57 | 8 | 2 | 64 | 427 | - | - | - | 29 | 92.3 | - | 16 | - | - | - |
| Kamel | 2021 | 555 | 466 | 84 | 5 | 64 | - | - | - | - | 16 | 95.5 | - | 9 | - | 4 | 9 |
| van der Sluis | 2015 | 108 | 78 | 20 | - | 62 | 381 | 340 | 18.5 | 17.6 | 26 | 95 | 1 | 16 | 33.3 | - | - |
| Washington | 2019 | 18 | 14 | 4 | - | 61.9 | 168 | - | 5.6 | - | 14.2 | 94.4 | 1.7 | 9.9 | - | - | - |
| Na (Totally Robot-assisted minimally invasive esophagectomy) | 2019 | 144 | 1 | 139 | 4 | 65.3 | - | - | 6.25 | 6.3 | 48.1 | - | - | 14 | - | 0 | 1 |
| (Hybrid Robot-assisted minimally invasive esophagectomy) | " | 70 | 2 | 67 | 1 | 65.5 | - | - | 10 | 7.1 | 46.2 | - | - | 14 | - | 1 | 3 |
| Pointer | 2022 | 350 | 303 | 38 | 9 | 66.06 | 424.52 | 232.21 | 15.7 | 1.7 | 21 | 95.7 | - | 9 | 14.9 | 2.6 | - |
| Naffouje | 2019 | 53 | 49 | 4 | 0 | 63.85 | 449 | - | 14.6 | - | - | 85.4 | - | 7 | 7.3 | - | - |
| Coker | 2014 | 23 | 23 | - | - | 64 | 231 | 100 | 8.70 | - | 15 | 91.3 | - | 9 | 9 | 4 | - |
| Yang | 2022 | 326 | 0 | 326 | 0 | - | 248 | 200 | 9.8 | - | 29 | 95.1 | - | 9 | 11 | - | 0.3 |
| Shridhar | 2015 |  | 77 | 8 | 5 | 66 |  |  | 0 | - | - | 100 | - | - |  | - | 0 |
| (Neoadjuvant chemoradiotherapy) |  | 67 |  |  |  |  | 434 | 149 |  |  | 20.2 |  |  |  | 6 |  |  |
| (No- neoadjuvant chemoradiotherapy) |  | 22 |  |  |  |  | 427 | 153 |  |  | 21.7 |  |  |  | 13.6 |  |  |
| Xu | 2021 | 43 | 5 | 38 | 0 | 64 | 384.42 | 110.47 | 0 | 0 | 17.42 | 100 | 0 | 10 | 7 | 0 | - |
| Fujita | 2022 | 55 | - | - | - | 68.9 | 448.1 | 111.6 | 3.6 | - | 31.8 | - | - | 14 | 9 |  |  |
| Dezube | 2022 | 70 | - | - | - | 66 | - | - | - | 12.9 | - | - | - | 10 | - | - | - |
| Zhang | 2018 | 249 | 0 | 249 | 0 | 63.4 | 250.6 | 215.5 | 12.9 | 1.2 | 18.5 | 93.2 | - | 11 | 10 | - | 0 |
| Sayed | 2022 | 58 | 7 | 50 | 1 | 56 | 360 | 150 | 7 | 7 | 16 | 97 | 2 | 10.5 | 19 | - | 3.45 |
| Betzler | 2022 | 71 | 58 | 13 | - | 63.2 | 395 | 250 | 21.1 | - | 24 | 93 | 4 | 15 | - | - | - |
| Wang | 2018 | 24 | 3 | 20 | 1 | 63 | 352.5 | 120 | 8.33 | - | 19 | 100 | 1 | 11 | 8.3 | - | - |
| Goel | 2018 | 27 | 1 | 26 | - | 53.5 | 342.7 | 208.2 | 11.1 | 3.7 | 18 | 96.3 | 6.35 | - | - | - | - |
| Tagkalos | 2020 | 50 | - | - | - | 62 | 383 | 331 | 12 | - | 27 | 92 | 1 | 12 | 12 | 0 | 4 |
| Babic | 2022 | 76 | 58 | 18 | - | 63 | - | - | 7.9 | - | - | 92.1 | 3.2 | - | 8.5 | - | - |
| van der Sluis | 2021 | 100 | 72 | 15 | 13 | 61 | 415 | 311 | 8 | 4 | 29 | 92 | 1 | 11 | 12 | 1 | 3 |
| Hodari | 2015 | 54 | 46 | 3 | 5 | 65.1 | 362 | 74 | 6.82 | 2.27 | 16.2 | 100 | - | 12.9 | 13.6 | 2 | - |
| van der Horst | 2017 | 31 | 12 | 19 | 0 | 66 | 435 | 350 | 19.4 | 29 | 22 | 96.8 | 1 | 15 | 32.3 | 6 | - |
| van der Sluis | 2019 | 54 | 41 | 13 | - | 64 | 349 | 400 | 24.1 | - | 27 | 93 | 1 | 14 | 28 | 2 | 9 |
| Reinstaller | 2022 | 45 | 31 | 13 | 1 | 61.8 | 395 | - | 6.7 | 15.6 | 26.3 | 95.5 | 2.3 | 15.5 | 11.1 | 0 | - |
| Kulkarni | 2022 | 25 | 10 | 15 | - | 59.2 | 407.1 | 190.8 | 12 | 8 | 16 | 96 | 2 | 10 | - | - | 0 |
| Park | 2016 | 114 | 4 | 110 | - | 63.1 | 419.6 | 208.7 | 14.9 | 1.8 | 43.5 | 97.4 | 1 | 16 | - | - | 2.5 |
| Cerfolio | 2016 | 85 | 72 | 12 | 1 | 63 | 360 | 35 | 4.3 | 5.9 | 22 | 99 | - | 8 | - | 3.5 | 10.6 |
| Na | 2022 | 178 | 0 | 178 | 0 | 65.7 | - | - | 6.6 | 7.4 | 43.1 | 96.6 | - | 13 | 5.1 | 0.7 | 2.2 |
| Daiko | 2021 | 10 | 2 | 8 | 0 | 69 | 382 | 52 | 10 | - | 54 | 90 | 15 | - | 10 | - | - |
| Guerra | 2021 | - | - | - | - | - | - | - | - | - | - | - | - | - | - | - | - |
| (All robotic) |  | 76 | 52 | 21 | 3 | 67 | 555 | 115 | 13 | 4 | 35 | 100 | 2 | 10 | - | 3.9 | 7.9 |
| (Robotic Ivor-Lewis) |  | 45 | 41 | 1 | 3 | 65 | 570 | 75 | 16 | 7 | 40 | 100 | - | - | - | 2 | 4 |
| (Robotic McKeown) |  | 25 | 6 | 19 | 0 | 67 | 540 | 100 | 12 | 0 | 30 | 100 | - | - | - | 7 | 15 |
| (Robotic Transhiatal) |  | 6 | 5 | 1 | 0 | 79.5 | 455 | 50 | 0 | 0 | 28 | 100 | - | - | - | 0 | 0 |
| Ali | 2021 | 1543 | - | - | - | 63.61 | - | - | - | - | 16.6 | 96.2 | - | 12.1 | - | - | 7.86 |
| Zhang | 2019 | - | - | - | - | - | - | - | - | - | - | - | - | - | - | - | - |
| (Linear stapled side-to-side anastomosis) |  | 35 | 9 | 25 | 1 | 61.4 | 355.6 | 108.6 | 8.6 | 2.9 | 22.5 | 100 | 0.9 | 11.7 | 8.6 | 0 | 0 |
| (Circular stapled anastomosis) |  | 42 | 5 | 34 | 3 | 61.9 | 343.3 | 113.4 | 4.8 | 0 | 20.5 | 100 | 1.1 | 12.8 | 9.5 | 0 | 0 |
| Kamarajah | 2022 | 1659 | 1367 | 292 | 0 | - | - | - | - | - |  | 96.2 | - |  | - | 2.2 | 5.9 |
| Puntambekar | 2015 | 83 | 12 | 67 | 4 | - | 204.9 | 86.8 | 3.61 | 1.2 | 18.4 | 97.6 | 1 | 10.4 | 1.2 | - | - |
| Chouliaras | 2022 | 67 | 60 | 7 | 0 | 63.5 | 511 | 100 | 3 | 1.5 | 20 | - | - | 7 | 6 | - | - |
| Hoelzen | 2022 | 51 | 45 | 6 | 0 | - | 513 | 0 | 23.5 | - | - | 96.1 | 3 | 18 | 21.6 | 0 | - |
| Chiu | 2017 | 20 | 3 | 17 | 0 | 64.2 | 499.5 | 355.7 | 15 | 5 | 18.2 | 100 | 1.7 | 13 | 5 | 5 | - |
| Egberts | 2022 | 220 | 179 | 34 | 7 | 64 | 425 | 200 | 13.2 | 1.36 | 25 | 92.9 | 3 | 15 | 19.5 | - | 3.6 |
| Chinnusamy | 2019 | 15 | 3 | 12 | 0 | 62.9 | 558 | 145 | 0 | 6.67 | 26 | - | - | 9 | 13.3 | - | 0 |
| Huang | 2020 | 11 | 1 | 9 | 1 | 57.09 | - | 598.2 | 9.09 | - | 38.6 | - | 6.55 | 23.5 | 9.09 | - | - |
| Galvani | 2008 | 18 | 18 | 0 | 0 | 54 | 267 | 54 | 33.3 | - | 14 | - | 1.8 | 10 | 11 | 0 | - |
| Meredith | 2018 | 147 | 126 | 21 | 0 | 66.4 | 415.4 | 158.3 | 2.7 | 3.4 | 20.4 | 100 | 2 | 9 | 6.8 | 0.68 | 1.4 |
| Defize | 2021 | 24 | 1 | 23 | 0 | 63 | 394 | 250 | 25 | 8.33 | 27 | 92 | 1 | 14 | 45.8 | 4.17 | - |
| Yang | 2020 | 280 | 0 | 280 | 0 | 63.1 | 244.1 | 211.4 | 11.8 | 1.5 | 19.3 | 93.9 | 2 | 11 | 8.9 | - | 0 |
| Booka | 2021 | 35 | 11 | 23 | 1 | 67 | 561 | 150 | 8.6 | 0 | 16 (3 field), 19 (2 field) | - | - | 24 | 14.3 | - | - |
| He | 2018 | 27 | - | 23 | 4 | 61 | 349 | 118 | 11.1 | 0 | 20 | - | - | 13.8 | - |  | 0 |
| Morimoto | 2021 | 22 | 1 | 21 | - | 67 | 507 | 107 | 4.6 | - | 27 | 82 | 23 | - | 9 | 0 | - |
| Mori | 2016 | 22 | 0 | 20 | 2 | 64 | 524 | 385 | 18.2 | 4.5 |  | 95.5 |  | 18 |  |  |  |
| Weksler | 2017 | 581 | 455 | - | 126 | 63 | - | - | - | - | 16 | 95.2 |  | - | - | 5.4 | 7.8 |
| Cerfolio | 2013 | 22 | 18 | 4 | - | 66 | 367 | 40 | 9.09 | 4.54 | 18 | 100 | - | 7 | - | - | - |
| Peri | 2022 | 12 | 7 | 1 | 4 | 69.3 | 467 | - | 0 | 16.7 | 32.5 | 100 | - | 13 | - | - | - |
| Kernstine | 2007 | 14 | 8 | 4 | 2 | 67 | 666 | 400 | 14 | - | 18 | 100 | - | - | 21 | - | - |
| (Thoracic phase robotic-assisted esophagectomy) |  | 26 | 0 | 25 | 1 | 64.2 | 385.5 | 140 | 7.7 | 0 | 17.4 | - | - | - | 7.7 | 3.8 | 0 |
| (Robotic-assisted thoracic esophagectomy) |  | 46 | 0 | 45 | 1 | 61.3 | 300 | 132.5 | 8.6 | 4.3 | 22.6 | - | - | - | 6.5 | 0 | 2.2 |
| Park | 2017 | 115 | 0 | 115 | 0 | 63.2 | - | - | - | - | 49 | 95.7 | - | - | 11.3 | - | - |
| Kim | 2010 | 21 | 1 | 20 | 0 | 61.5 | 410 | 150 | 19.1 | - | 38 | 95.2 | 2 | 21 | - | - | 0 |
| Somashekhar | 2017 | 35 | 9 | 26 | 0 | 61 |  |  | 0 | 0 | 32 | 100 | 1.23 | 8 |  | 0 |  |
| (Initial 10 cases) |  | - | - | - | - | - | 429.2 | 433.2 |  |  |  |  |  |  |  |  |  |
| (Subsequent 25 cases) |  | - | - | - | - | - | 321.1 | 256.3 |  |  |  |  |  |  |  |  |  |
| Keeney-Bonthrone | 2022 | 87 | 80 | 7 | 0 | 66.3 | 292 | - | 17.2 | 4.6 | 13.3 | - | - | 7 | 12.6 | 3.4 | - |
| Chao | 2020 | 39 | 1 | 38 | 0 | 57.4 | - | 83.3 | 12.3 | 0 | 29 | 94.9 | 0.85 | - | 2.6 | 0 | 0 |
| Konstantinidis | 2020 | 346 | - | - | - | 64 | - | - | - | - | 16 | 95 | - | 9 | - | - | 6 |
| Osaka | 2018 | 30 | - | - | - | 62 | 563 | 197 | 10 | - | - | - | - | 17 | 6.7 | - | - |
| Yang | 2022 | 181 | 0 | 181 | 0 | 65 | 203.8 | 200 | 12.2 | 2.8 | 23 | 95 | 1 | 9 | 9.9 | 0 | 0.6 |
| van der Horst | 2023 | 20 | 13 | 7 | 0 | 64 | 506 | 425 | 30 | 25 | 42 | 95 | 1 | 17 | 40 | 0 | 0 |

Twelve studies carried out sub-group analyses; the types of sub-group are specified in brackets.

**Supplementary Table 1. All data from included studies.**

**Supplementary Figure 1. Newcastle-Ottawa Assessment of Included Studies**


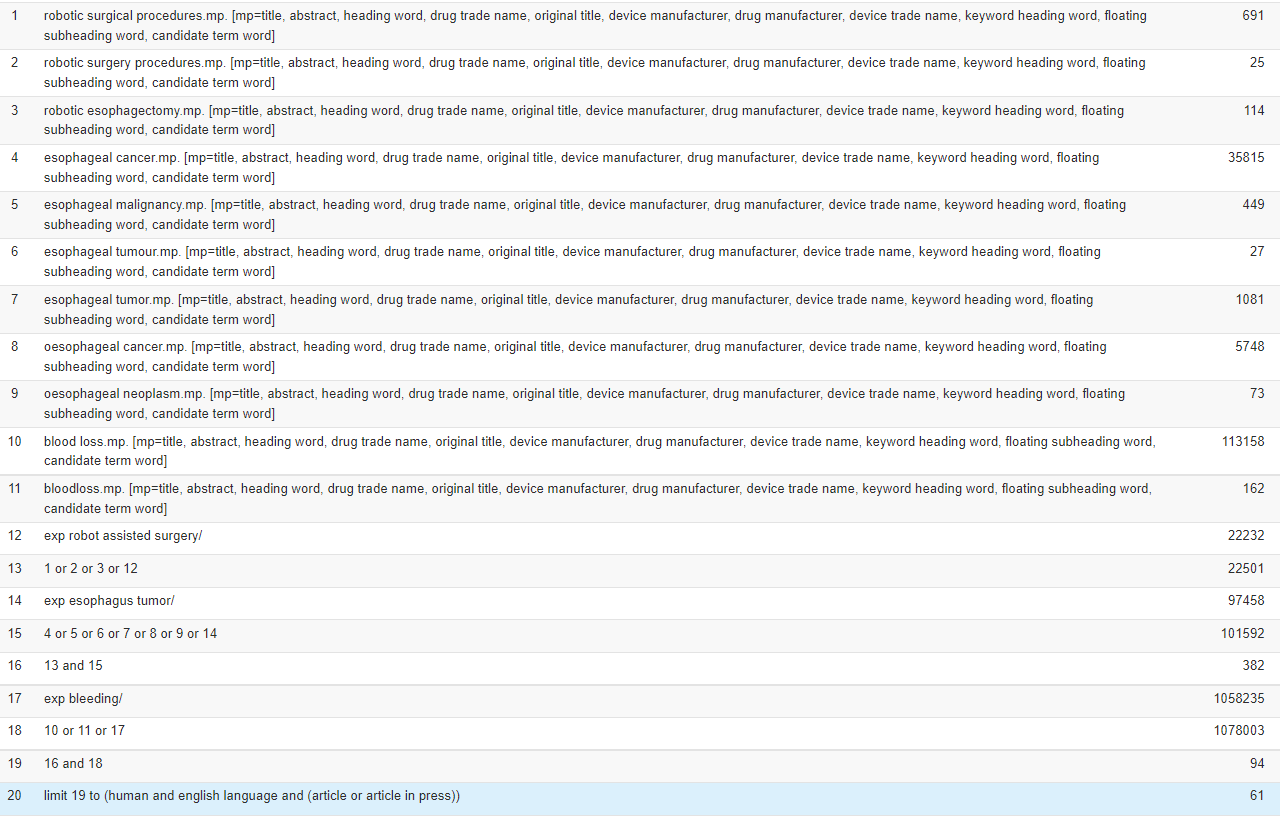


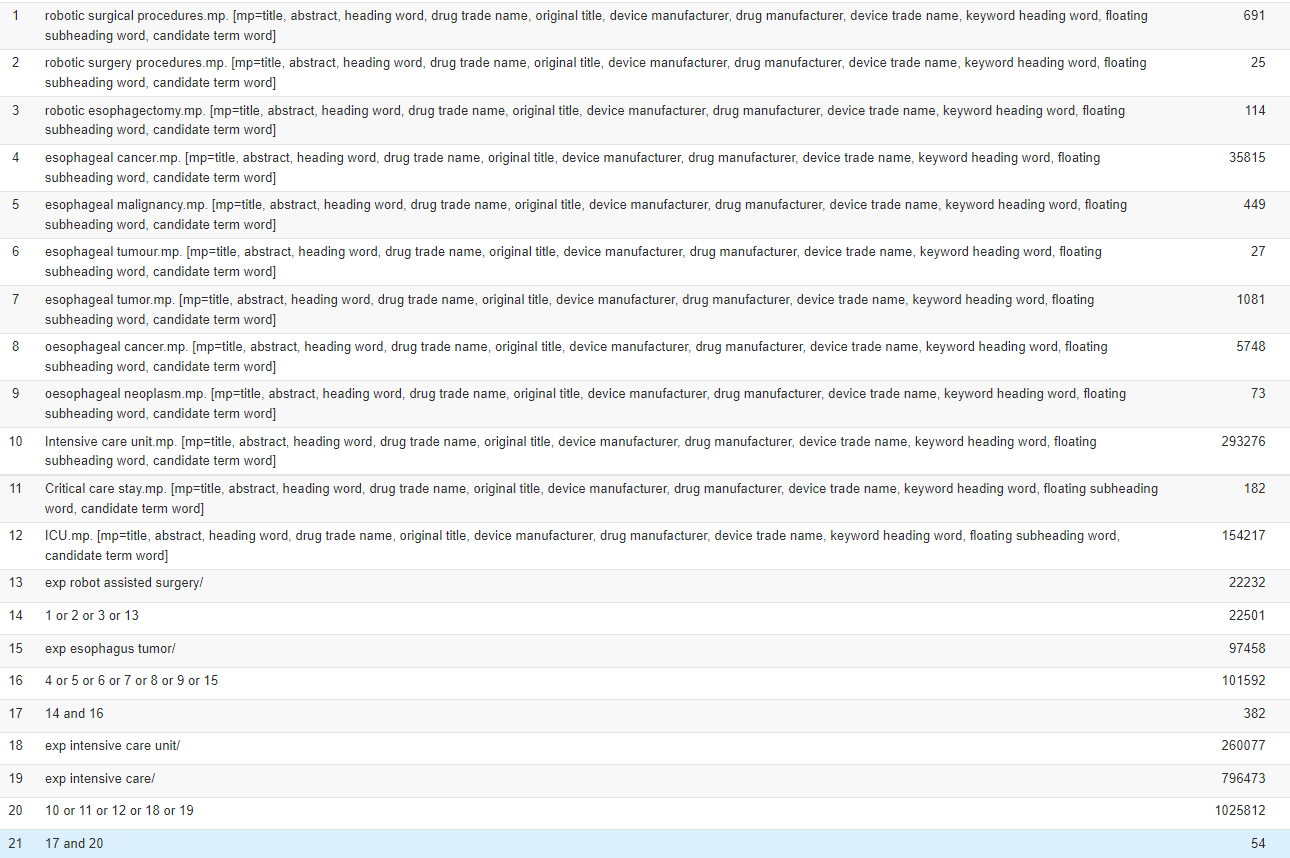


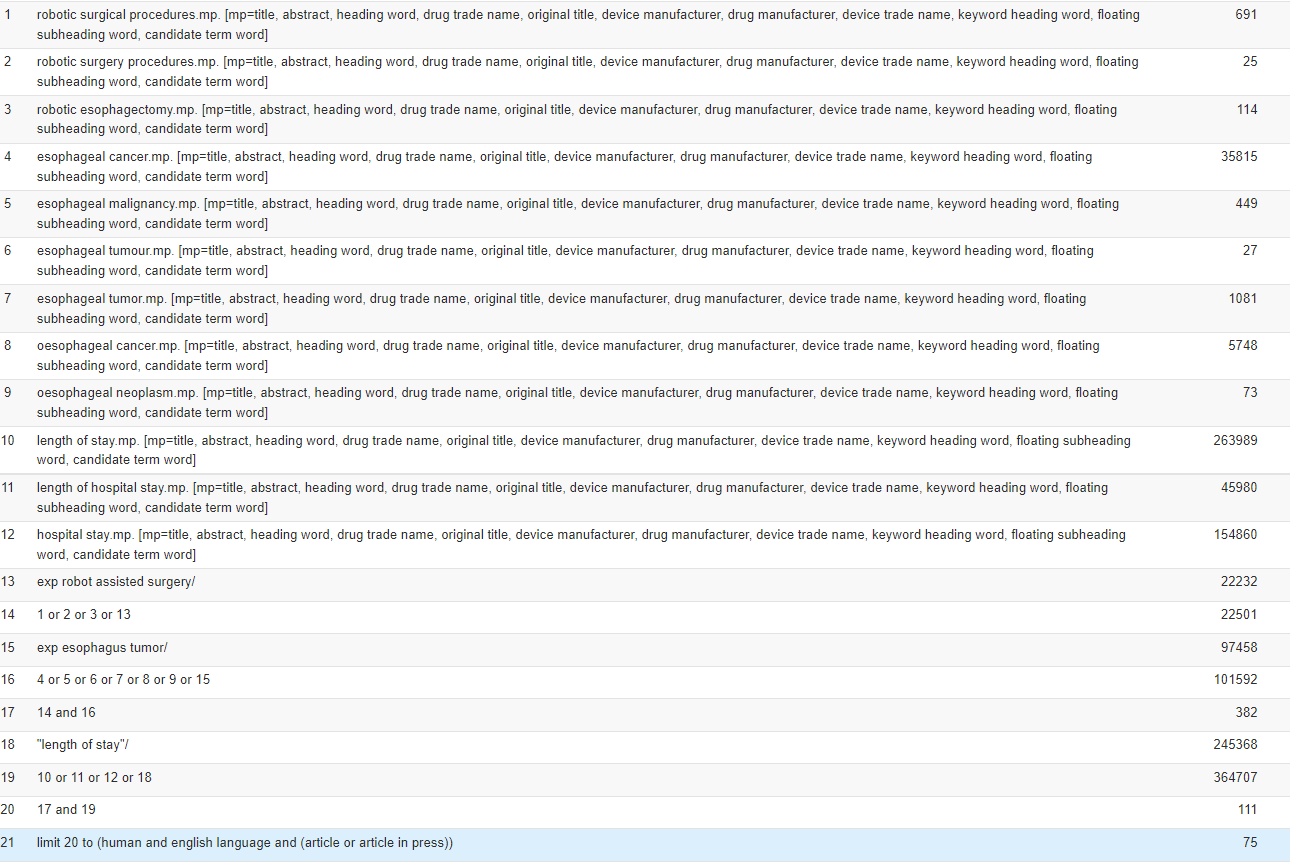


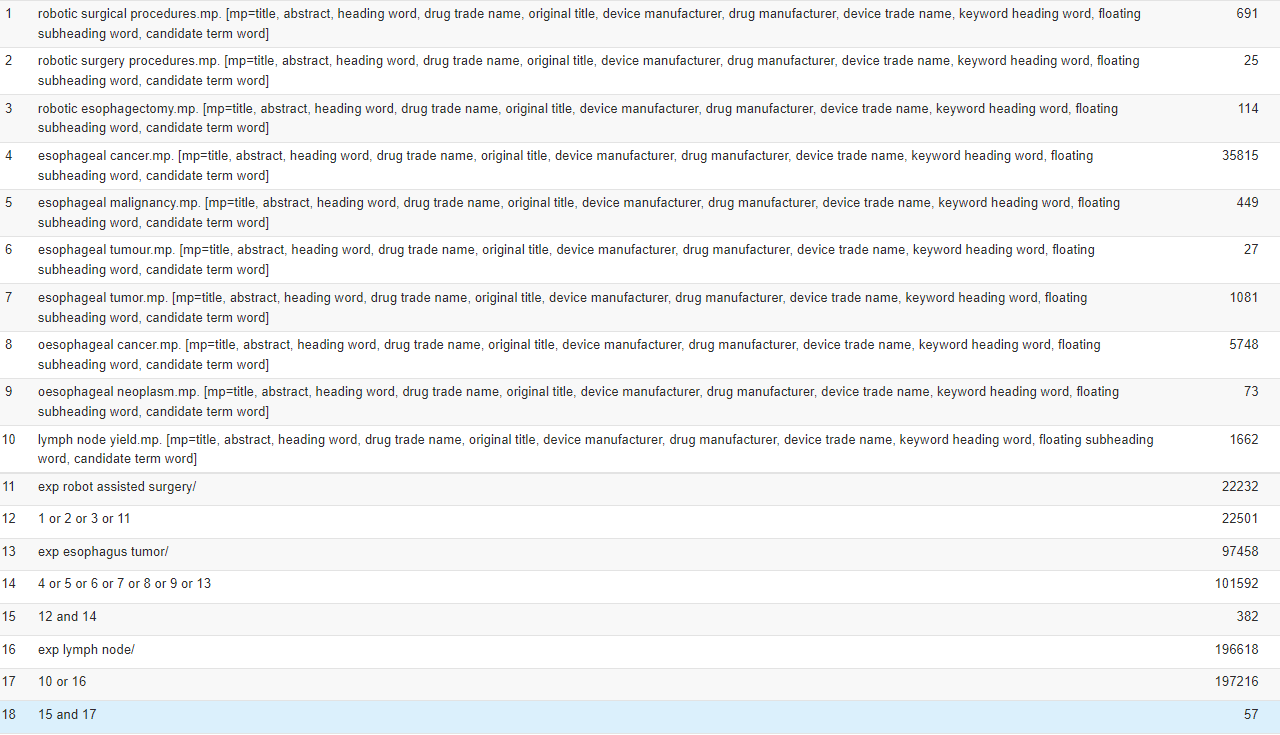


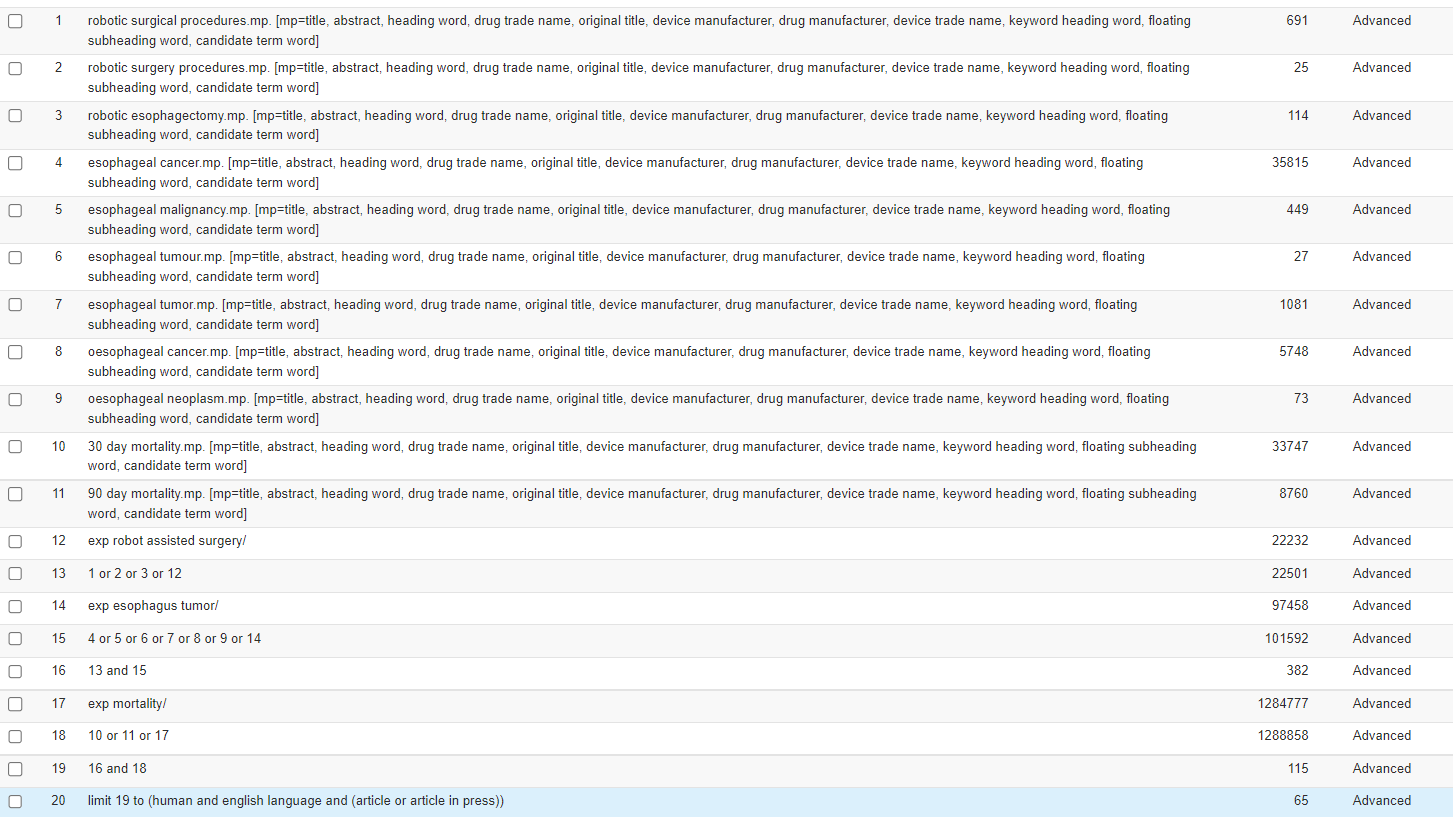


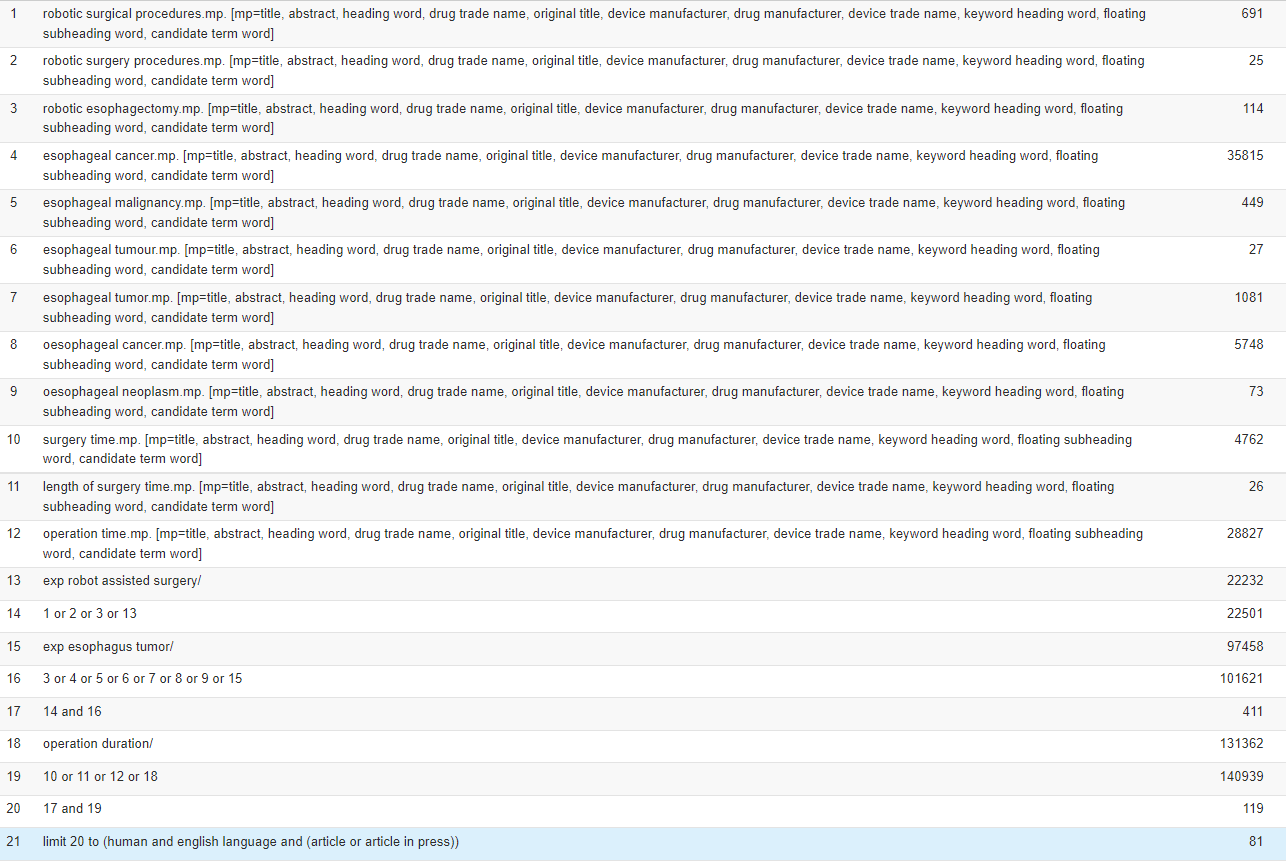


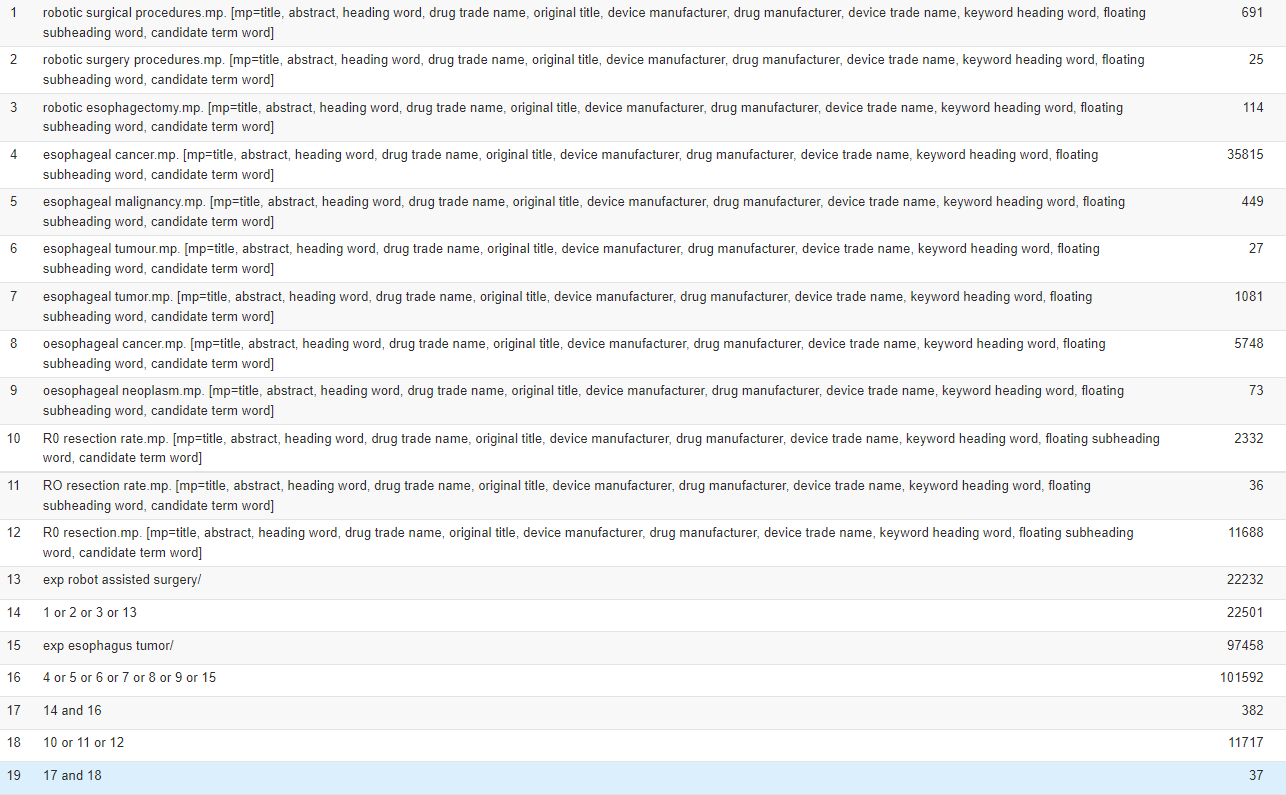


**Supplementary Table 2. Searched keywords and results on Embase**

**
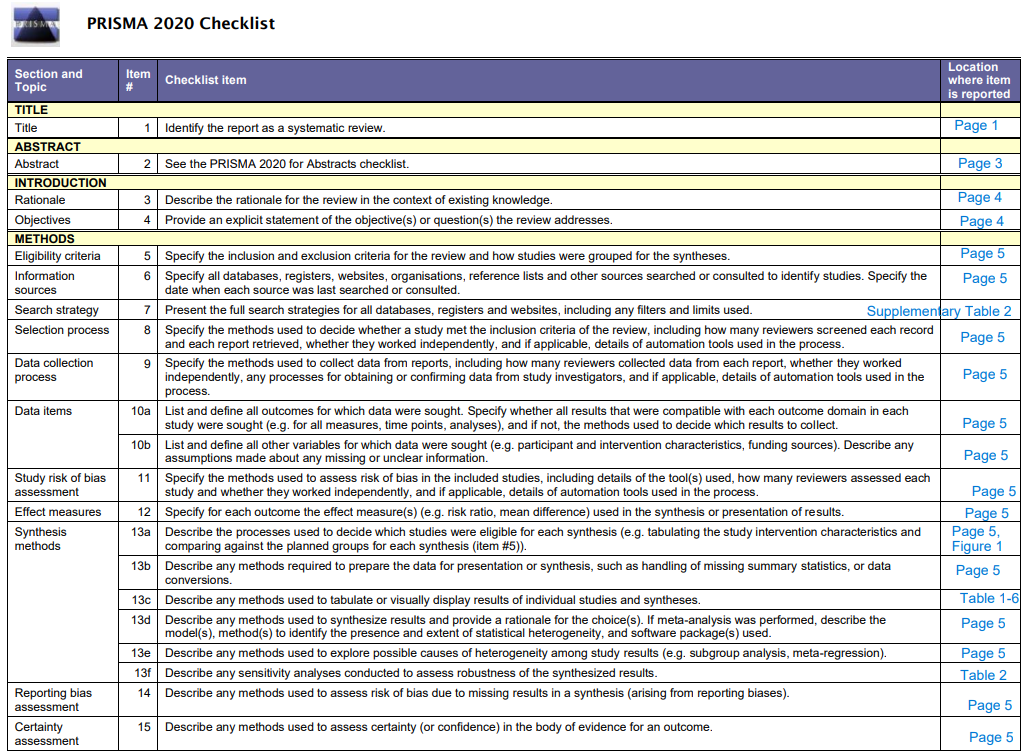
Supplementary Table 3. PRISMA checklist**

**
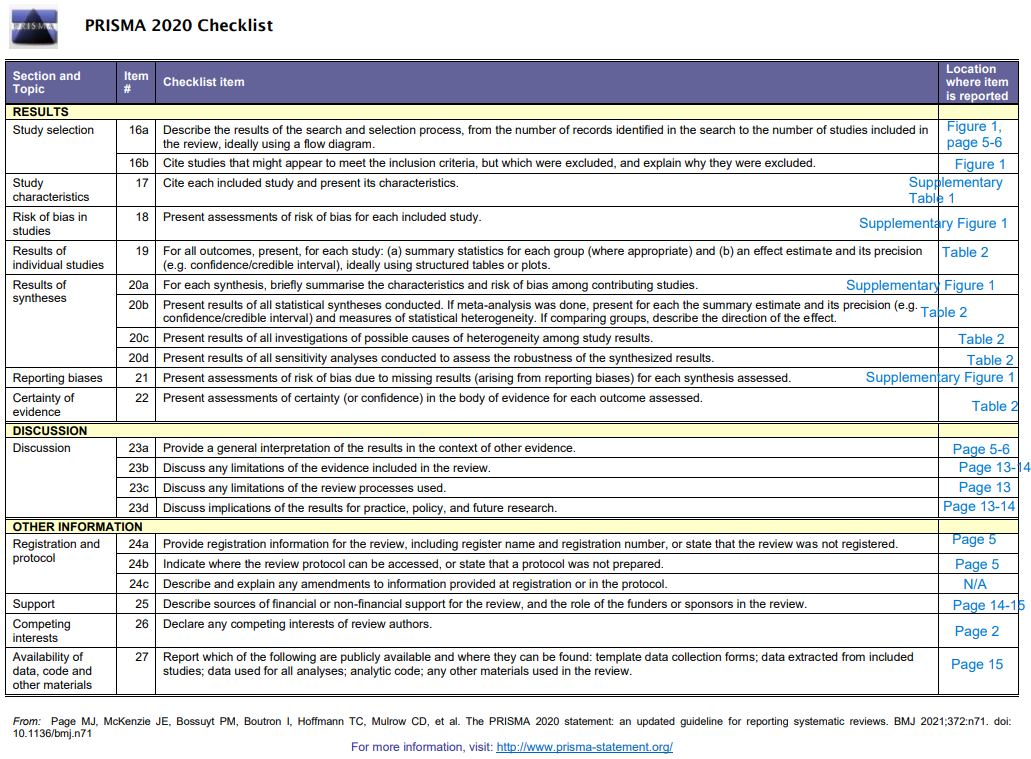
**

**Operative time (minutes)**

**
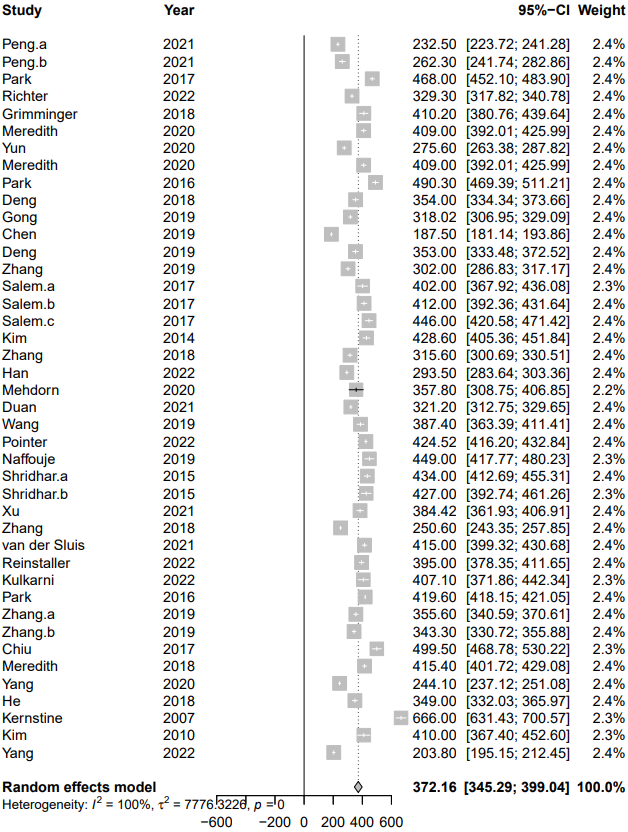
**

**Supplementary Figure 2. Forest plot on operative time**

**Estimated blood loss (mLs)**

**
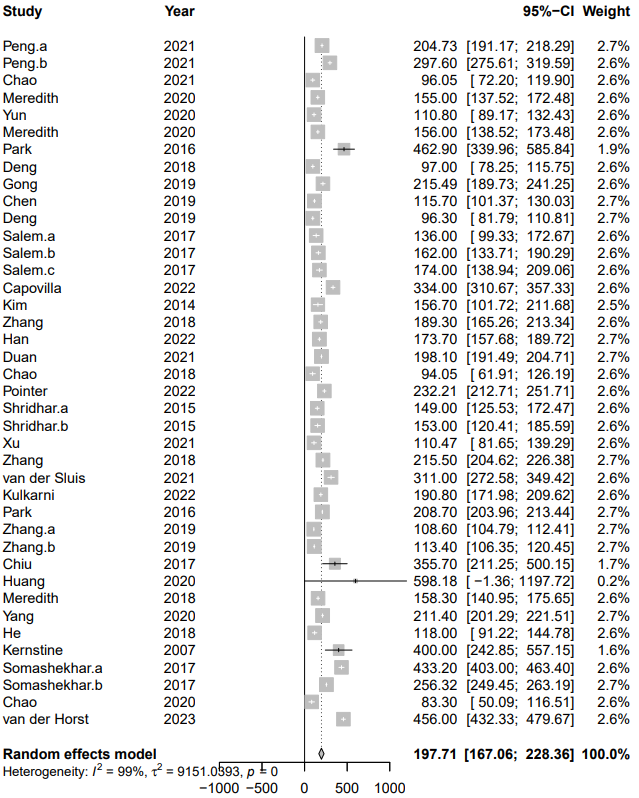
**

**Supplementary Figure 3. Forest plot on estimated blood loss**

**Critical Care Length of Stay (days)**

**
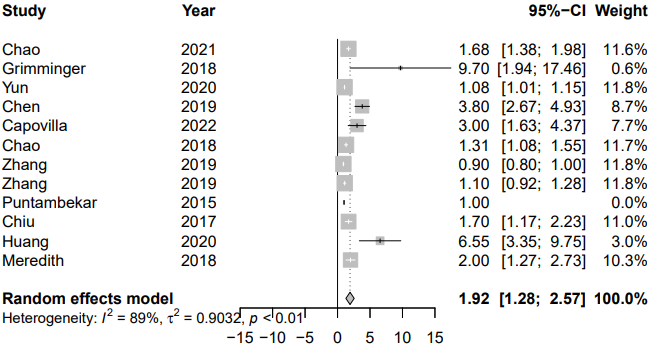
**

**Supplementary Figure 4. Forest plot critical care length of stay**

**Total inpatient Length of Stay (days)**

**
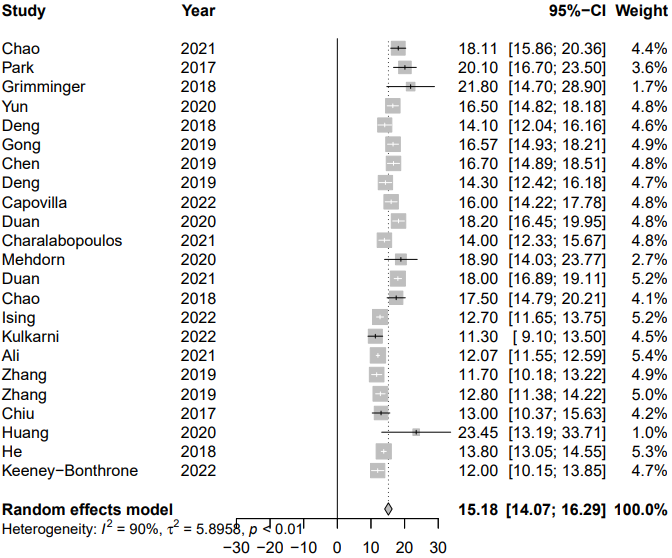
**

**Supplementary Figure 5. Forest plot on total inpatient length of stay**

**Chyle Leak**

**
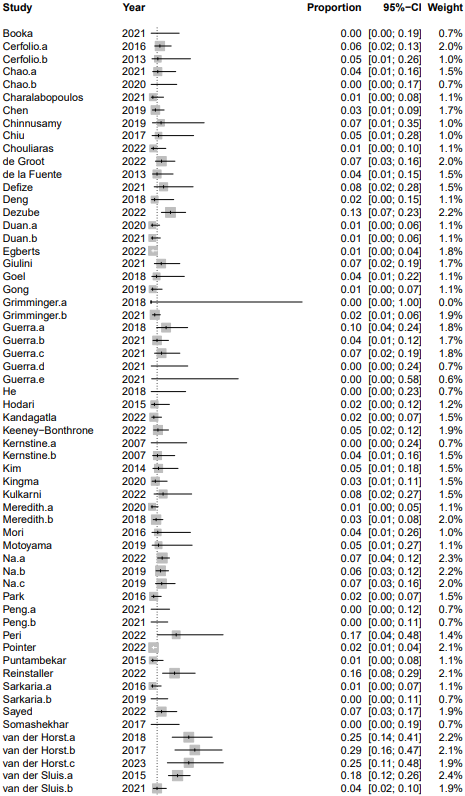
**

**Chyle Leak (continued)**


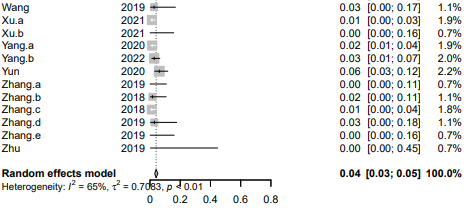


**Supplementary Figure 6. Forest plot on chyle leak**

**Post-operative pneumonia**

**
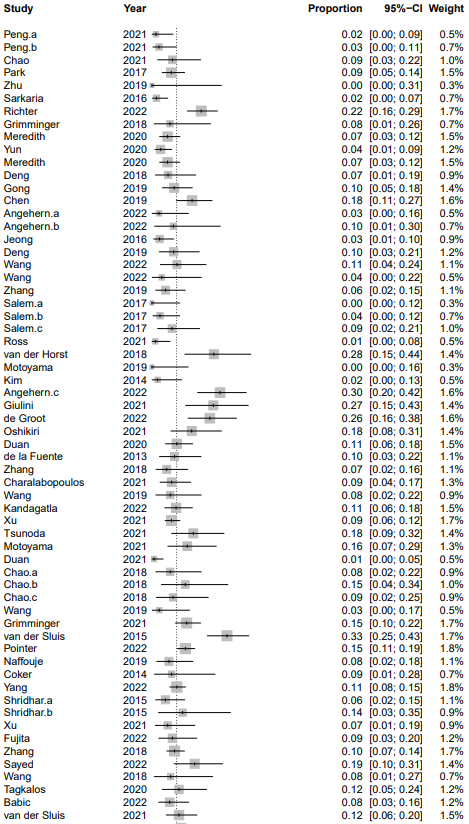
**

**Post-operative pneumonia (continued)**

**
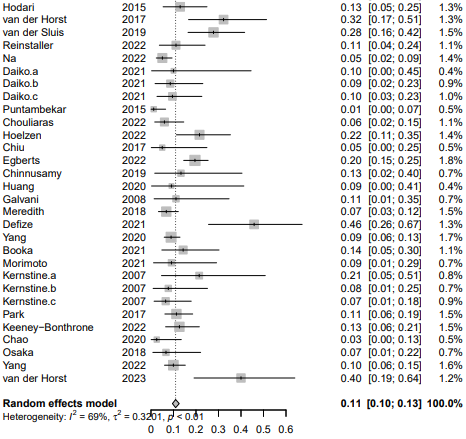
**

**Supplementary Figure 7. Forest plot on post-operative pneumonia**

**Lymph Node Yield**

**
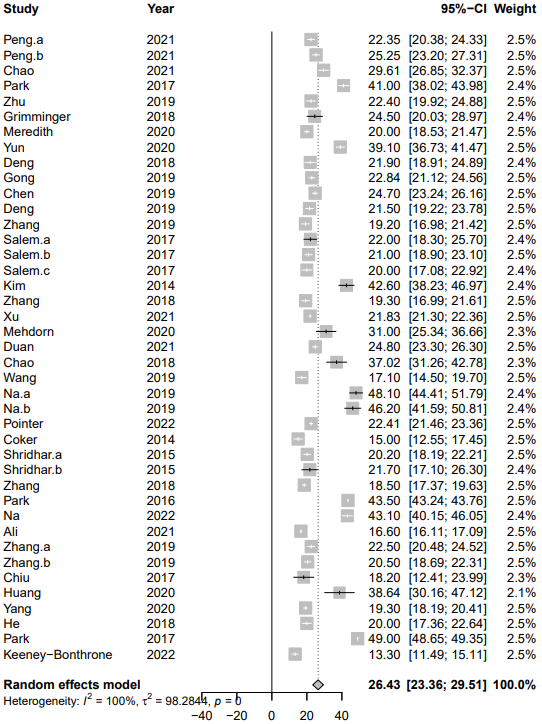
**

**Supplementary Figure 8. Forest plot on lymph node yield**

**R0 resection rate**

**
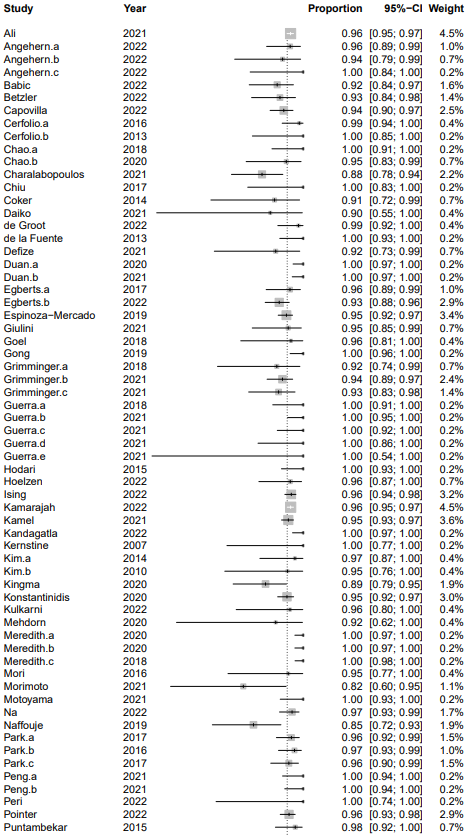
**

**R0 resection rate (continued)**

**
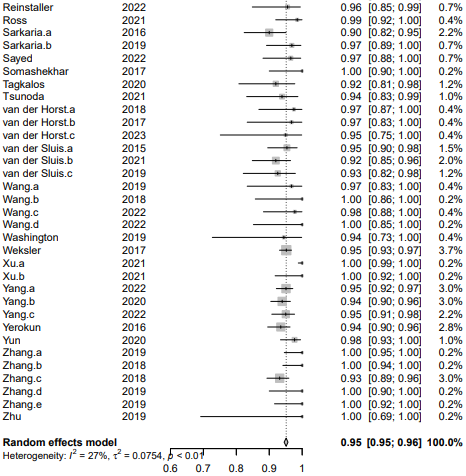
**

**Supplementary Figure 9. Forest plot on R0 resection rate**
